# Genome-wide characterization of copy number variants and their functional relevance in indigenous draught cattle of South Asia

**DOI:** 10.1371/journal.pone.0353468

**Published:** 2026-07-13

**Authors:** Tafara Kundai Mavunga, Johann Sölkner, Gábor Mészáros, Maano Bryton Malima, Rudolf Pichler, Saravanan Ramasamy, Loku Galappattige Sampath Lokugalappatti, Ei Thandar, Menghak Phem, Farai Catherine Muchadeyi, Yosra Ressaissi, Mario Barbato, Kathiravan Periasamy

**Affiliations:** 1 Animal Production and Health Laboratory, Joint FAO/IAEA Centre of Nuclear Techniques in Food and Agriculture, Department of Nuclear Sciences and Applications, International Atomic Energy Agency, Vienna, Austria; 2 Institute of Livestock Sciences, Department of Agricultural Sciences, Universität für Bodenkultur Wien, Vienna, Austria; 3 Agriculture Research Council, Onderstepoort, Pretoria, South Africa; 4 South African Medical Research Council Genomics Platform, Tygerberg, South Africa; 5 Tamil Nadu Veterinary and Animal Sciences University, Chennai, India; 6 Faculty of Veterinary Medicine and Animal Science, University of Peradeniya, Peradeniya, Sri Lanka; 7 Livestock Breeding and Veterinary Department, Ministry of Agriculture, Livestock and Irrigation, Yangon, Myanmar; 8 National Animal Health and Production Research Institute, General Directorate of Animal Health and Production, Ministry of Agriculture, Forestry and Fisheries, Phnom Penh, Cambodia; 9 Dipartimento di Scienze Veterinarie, Università degli Studi di Messina, Messina, Italy; Chattogram Veterinary and Animal Sciences University, BANGLADESH

## Abstract

Copy number variations (CNVs) are an important source of structural genomic variation and contribute to phenotypic diversity in cattle, including traits related to production, reproduction, and adaptation. In this study, we performed a genome-wide characterization of copy number variation regions (CNVRs) in Asian zebu cattle representing eight indigenous draught breeds traditionally used for ploughing, wet-field agriculture, and carting. CNVs were detected using two read-depth–based approaches, CNVnator and CNVcaller, which identified 7,705 and 5,640 CNVs, respectively. Integration of results from both methods using a 50% reciprocal overlap criterion yielded 6,143 CNVRs. The average number of CNVRs per breed ranged from 4,607–5,005, collectively covering approximately 6.23% of the autosomal genome. Of these, 2,697 CNVRs were shared across all breeds, whereas 190 CNVRs were breed specific. Population differentiation based on CNVs, estimated using pairwise V_ST_ statistics, indicated that the Hallikar breed exhibited the highest average differentiation (0.07) relative to other breeds. A total of 4,868 genes overlapped with the identified CNVRs and were enriched for biological processes associated with immune regulation, metabolic function, and adaptive responses. Further comparison with cattle quantitative trait loci identified 65 unique QTLs, predominantly linked to carcass, fertility, reproduction, and growth traits. Overall, this study describes the genome-wide distribution and diversity of CNVRs in South Asian indigenous draught cattle and provides baseline genomic information for further investigations into structural variations relevant to breeding, management, and conservation.

## 1. Introduction

Copy number variants (CNV) in livestock have gained increasing attention in the recent years due to their role in genomic diversity and phenotypic expressions of economically important traits [1]. CNVs are heritable structural variants characterized by deletions or duplications of DNA segments ranging in size from ~1 kilobase (Kb) to several megabases (Mb) [2]. CNVs span a larger proportion of the genome and arise from mechanisms such as unequal recombination, replication errors or genomic rearrangements [3,4]. These repeated genomic segments vary between individuals in a population and contribute to genetic and phenotypic variation. The differences in the copy number of genes result in changes in the gene expression either due to alterations in gene dosage or disruption effect by deletion, duplication, inversion and translocation of DNA sequence [5–7]. CNVs are an important source of variation, and their position largely determines the effect; those located in coding regions alter the protein function, whilst those in regulatory regions alter the level of gene expression [8–10]. Single nucleotide polymorphisms (SNPs) have been routinely used to assess population genetic diversity and genomic predictions for production related traits in cattle. Recent studies [11,12] have indicated SNP markers alone are not sufficient to predict complex traits efficiently and CNVs provide significant additional information to improve the accuracy.

In livestock, several studies have used array-based methods for detection of CNVs [13–17]. However, these approaches have methodological limitations such as limited genomic coverage, low resolution, hybridization noise, limitations in detection of CNVs less than 1kb size and incorrect identification of CNV boundaries [18,19]. Advancement in high throughput sequencing and increased access to next generation sequencing technology has led to improved resolution, sensitivity and the development of new strategies to identify CNVs [20,21]. Whole genome sequencing (WGS) enables the detection of structural variants by identifying abnormal alignment patterns that indicate potential genomic rearrangement breakpoints. Four main approaches are commonly used namely, read pair (RP), split reads (SR), read depth (RD) and assembly based (AS) [18,22–25]. Although each of these methods have been reported to be efficient in detecting CNVs [18], combining one or more approaches can be advantageous in reducing the false positives and improving the accuracy. Various studies have reported the use of these methods in investigating the association between CNVs in specific genes and various phenotypic traits in livestock. For instance, CNVs have been linked to adaptive traits in cattle [26,27], growth performance in sheep [28–30], goats [31,32] and buffaloes [21], indicating their functional significance and impact on economic traits in livestock production.

The zebu cattle breeds of South Asia exhibit extensive genetic diversity in terms of phenotypes and varied utilities such as milk, draught, meat, dung, etc., apart from their adaptability to hot and arid production environments, resistance or tolerance to tropical vectors and diseases [33,34]. Such extensive genetic variations have been shaped by years of adaptation to a wide range of environmental and management conditions and for specific purposes such as draught power [35]. Draught breeds such as Hallikar, Kangayam, Bargur and Deoni in India, Sri Lankan White Cattle, Pyar Zein and Shwe Ni from Myanmar, and Kdarm Red from Cambodia are valued for their strength and draught power, making them ideal for agricultural activities such as ploughing and transportation [36–41]. Hallikar cattle (Karnataka, South India) are known as sturdy and powerful draught animals [36], Kangayam cattle (Tamil Nadu, South India) are known for their ability to work for long hours [42] and Bargur cattle (Tamil Nadu, South India) are adapted to hilly terrain but are relatively temperamental in nature and difficult to train [43,44]. Deoni cattle (Maharashtra, Western India) are a dual-purpose breed used for both draught and milk, and are medium heavy animals found in three-colour variations viz Wannera, Balankaya and Shevera [45]. White Cattle of Sri Lanka are reared inside the forest areas of the Eastern province and are primarily used for draught purposes [46]. Additionally, draught breeds such as Pyar Zein (central and southern regions of Myanmar) and Shwe Ni (central dry zone of Myanmar) are well-suited for wet-field operations, particularly in paddy cultivated areas. Lastly, Kdarm Red from Cambodia (also known as Kor Khmer or Gor Srok) is a dual-purpose breed, primarily used for draught power, but with moderate potential for growth and meat production. The specialized roles of zebu cattle reflect their development in diverse agro-ecological systems, emphasizing the necessity to study the underlying genomic variations that contribute to their adaptation and production potential.

CNVs in cattle have been identified using a range of approaches, including early array-based techniques such as array comparative genomic hybridization (array CGH), quantitative PCR, and fluorescence in situ hybridization [47], as well as more recent high-resolution methods based on SNP arrays and next-generation sequencing (NGS) [48,49]. NGS-based approaches, including read depth, paired-end mapping, and split-read strategies, have improved genome-wide detection and resolution of CNVs [50], enabling population scale analyses across multiple breeds. Despite these advances, the current understanding of CNVs in Asian zebu cattle remains limited. Many studies have focused on a relatively small number of breeds [51–53] or have been biased towards taurine or globally distributed populations such as Nellore, Brahman and Gir [54,55], leading to underrepresentation of indigenous South and Southeast Asian zebu cattle [56]. In addition, inconsistencies in CNV detection methods, reliance on taurine reference genomes, and variability in sequencing depth can affect the accuracy and comparability of results across studies. Consequently, there is a lack of comprehensive, comparative analyses capturing CNV diversity across genetically distinct zebu populations in the region. To address this gap, the present study investigates genome-wide CNV patterns in selected breeds representing South and Southeast Asian zebu cattle. Investigating the role of CNVs in breed differentiation, and adaptation to local agro-ecological conditions may reveal valuable insights for developing sustainable breeding strategies and promoting effective utilization of these native cattle populations. The present study aims to provide new insights into structural genomic variation by conducting a comprehensive whole genome characterization of CNVs in eight South and Southeast Asian zebu cattle populations using the read depth approach and to identify structural variants intersecting key genomic regions associated with production and adaptation traits.

## 2. Materials and methods

### 2.1. Animal ethics statement

No animals were specifically sampled for the present study. All genome sequences reported in the study were derived from existing DNA/sample repositories available at Animal Production and Health Laboratory, Joint FAO/IAEA Centre of Nuclear Techniques in Food and Agriculture, International Atomic Energy Agency, Vienna, Austria. As no live animals were handled and no new samples were collected, ethical approval was not required for this study.

### 2.2. Inclusivity in global research

Additional information regarding the ethical, cultural, and scientific considerations specific to inclusivity in global research is included in the S1 File.

### 2.3. Genome resequencing

Whole genome resequencing (WGS)of 40 cattle from eight distinct breeds was performed with five samples per breed, the details of which are shown in Table 1. All WGS data generated in the study are available in the form of paired end raw sequences (fastq.gz format) at NCBI under the BioProject accession number PRJNA1358578 (https://www.ncbi.nlm.nih.gov/bioproject/?term=PRJNA1358578). As indicated under the Ethics Statement, all the DNA samples used in the present study were derived from the existing Genetic Repository at Animal Production and Health Laboratory, Joint FAO/IAEA Centre of Nuclear Techniques in Food and Agriculture, International Atomic Energy Agency, Vienna, Austria. The DNA available at the FAO/IAEA repository were extracted from blood samples collected by jugular venipuncture into EDTA vacutainer tubes and isolated using MasterPure DNA Purification Kit (Biozym, Illumina Inc, USA). It was ensured that the samples included in the study were collected from unrelated cattle using the information available in the Genetic Repository module of FAO/IAEA Genetic Laboratory Information and Data Management System (GLIDMaS). The DNA samples were subjected to quality control using Nanodrop 2000 spectrophotometer (ThermoScientific, USA) initially to estimate concentration, followed by Quant-iT™ PicoGreen™ assay to ascertain the quantity of good quality double strand DNA. Whole genome resequencing was performed using Illumina Novaseq 6000 using paired end 150 bp reads with a target coverage of ~20x per sample at Neogen Europe Ltd., Ayrshire, Scotland.

**Table 1:** Description of samples used in the study.

Country	Breed	Number of samples	Type
India	Bargur	5	draught
	Deoni	5	draught/milk
	Hallikar	5	draught
	Kangayam	5	draught
Sri Lanka	White Cattle	5	draught
Cambodia	Kdarm Red	5	draught/meat
Myanmar	Pyar Sein	5	draught
	Shwe Ni	5	draught

### 2.4. Quality control of raw sequence reads and alignment

Raw sequencing reads in FASTQ format were initially assessed using FastQC v0.11.9 [57] to evaluate per-base quality scores, GC content and adapter contamination. Reads were then pre-processed with fastp v0.23.2 [59] to remove adapter sequences, trim low-quality bases (Phred <20), and discard short reads (<50 bp), with all other parameters set to default. Post-trimming quality was reassessed using FastQC to confirm improvement in read quality and removal of adapter contamination. High-quality reads were aligned to the indexed Zebu cattle reference genome NIAB_ARS_BosIndicus_Tharparkar_1.0 (NCBI RefSeq assembly, GCF_029378745.1) [58] using BWA-MEM v0.7.17 [59] with default parameters. The average mapping rate was 99% and the average sequencing coverage was 21.2 × . The alignment output files in sequence alignment map (SAM) format were sorted, indexed, and converted to binary alignment map (BAM) format using sequence alignment/map tools (SAMtools v1.9) [60]. The potential duplicate reads were marked using Picard tools (https://broadinstitute.github.io/picard). The resulting BAM files with marked duplicates were used as input for CNV calling.

### 2.5. CNV and CNVR detection

A read depth approach was used to detect copy number variations. To increase the reliability of CNV detection, CNVnator [61] and CNVcaller [62] were used. CNVnator utilizes fixed-bin read-depth histograms for sensitive detection of CNVs whereas CNVcaller applies population-based normalization and segmentation models to improve breakpoint resolution and reduce false positives. CNV detection using CNVnator was carried out for the autosomal chromosomes only, using a bin size of 100 bp, following the recommendations of Abyzov et al., [61]. The following steps were run; CNVnator -tree, CNVnator -his, CNVnator -stat, CNVnator -partition and CNVnator -call and identification of CNV occurrence in the preset window was done. The software calls CNVs per individual and to enhance the CNV prediction quality control, raw CNVs were filtered by retaining only those with q0 < 0.5, size >1kb and p-value<0.01 based on t-test statistics. A q0 filter removes any CNV calls with q0 > 0.5 and the metric represents the fraction of reads within a region that have zero mapping quality which means reads with multiple mapping locations in the genome. This is crucial because regions with q0 > 0.5 are likely enriched for ambiguously mapped reads that potentially lead to false positive CNV detection. To compare the results from CNVcaller, individual CNVs were merged using 50% reciprocal overlap to create population based CNVRs.

CNVcaller is a population-based CNV detection software, that analyses population sequencing data across individuals simultaneously rather than independently. This approach leverages on using population-level read depth information to perform local depth normalization and segmentation across samples (adaptive binning instead of fixed bin size used in CNVnator), resulting in reduced false positives and improved breakpoint accuracy. Initially, the reference genome used in the present study (NIAB_ARS_BosIndicus_Tharparkar_1.0) lacked the duplicated window record files required for CNVcaller’s absolute number correction. Hence, custom duplicated window record files were generated following the official CNVcaller pipeline (https://github.com/JiangYuLab/CNVcaller). Briefly, the reference database for *Bos indicus* was created using NIAB_ARS_BosIndicus_Tharparkar_1.0 which was segmented into into k-mer sequences (overlapping windows) using the CNVcaller script 0.1.Kmer_Generate.py with a recommended window size of 800 bp [62] to optimize CNV detection and boundary resolution. The resulting k-mer FASTA file was then aligned back to the reference genome using BLASR after constructing the suffix array index with sawriter. BLASR alignment was performed using the parameters recommended in the CNVcaller documentation for sensitive k-mer mapping. Finally, duplicated window record files were generated from the BLASR alignment [63] output using 0.2.Kmer_Link.py, producing the genome-wide window linkage file required for downstream CNV analysis. The resulting reference database and duplicated window files were then used for CNV detection in CNVcaller. The custom scripts used for generating the CNVcaller duplicated window files and reference database are available in the GitHub (https://github.com/tafarakundai/cnvcaller-custom-reference-db). CNVcaller calculated the GC-corrected normalized read depth for each sample to standardize the copy number in each window and classify different genotypes. The following CNVcaller parameters were used for filtering: -f 0.1 to set the frequency threshold of 10% for downstream analysis, -h 3 to retain CNVs shared in at least three samples, and -r 0.5 to set the minimum reciprocal overlap to be 50% between CNVs for merging or creating population based CNVRs. Additionally, CNVs with a Silhouette score of >0.5 and those located in unplaced scaffolds were excluded from further analysis. It is important to note that the CNVcaller filtering thresholds (-f 0.1, -h 3) used in this study prioritize high confidence CNVRs and might have reduced sensitivity to rare or low frequency variants.

### 2.6. Population differentiation based on CNVR

V_ST_ of CNVRs between a pair of populations was calculated as V_ST_ = V_T_-V_S_/V_T_ where V_T_ is the total variance of copy numbers among two breeds and V_S_ is the average variance within each population, weighted by the number of individuals in the population. The pairwise differentiation among breeds was visualized using a heatmap generated by a custom python script.

### 2.7. Functional annotation and enrichment analysis of genes overlapping CNVRs

CNVRs identified by both CNVcaller and CNVnator were merged using 50% reciprocal overlap between regions. The merged CNVRs were annotated using ANNOVAR [64] with a custom reference gene database derived from NIAB_ARS_BosIndicus_Tharparkar_1.0 gene transfer format (GTF) file. This GTF file was converted into ANNOVAR refGene format using a custom Python script. ANNOVAR then produced gene-based annotations to identify overlapping transcripts with the CNVRs. Functional analysis of genes overlapping CNVRs for enrichment of Gene Ontology-Biological Process (GOBP) and Kyoto Encyclopedia of Genes and Genomes (KEGG) pathway was conducted using ShinyGO v0.82 [65]. The list of background gene set used to perform enrichment analysis is provided in the S2 File. A threshold of FDR < 0.01 and Fold Enrichment >2.5 was used to identify significantly enriched GOBP and KEGG pathways.

### 2.8. Identification of QTLs associated with CNVRs

The CNVRs detected across all populations as well as those shared among breeds were searched for overlap with Quantitative Trait Loci (QTL) reported in cattle using Animal QTL database, that consisted of 194,095 cattle QTLs or associations [66]. Regions overlapping with known QTLs, and their function associated with QTLs were identified.

## 3. Results

An average of 421.6 million paired end reads per sample was generated across different breeds of cattle. Evaluation of the quality of sequence data confirmed that all the investigated samples possessed sufficient depth and integrity for the detection of copy number variations. Following quality control and adapter trimming, approximately 99% of reads were successfully aligned to the *Bos indicus* reference genome (NIAB_ARS_BosIndicus_Tharparkar_1.0), indicating minimal alignment bias and high sequence quality. The mean genome-wide coverage was 21.2 × providing reliable CNV detection using read depth–based methods. The uniform sequencing depth across the samples is critical for precise CNV calling, and the narrow variation observed across the dataset reflects consistency in sequencing and data quality. A detailed summary of sequencing and alignment statistics for each sample is presented in Table 2.

**Table 2:** Sample-wise sequencing metrics of cattle investigated in the study.

Breed (Country)	Sample	Total Reads	Mapped Reads	Coverage
Bargur (India)	IBR1	434,047,178	99.30%	22.9
	IBR2	408,639,807	99.85%	21.0
	IBR3	399,606,295	99.89%	20.4
	IBR4	419,549,818	99.86%	21.2
	IBR5	425,540,206	99.88%	23.1
Deoni (India)	IDN1	434,140,502	99.90%	22.3
	IDN2	405,129,807	99.90%	20.6
	IDN3	478,578,091	99.75%	25.0
	IDN4	393,221,955	99.92%	19.7
	IDN5	379,314,501	99.87%	19.8
Hallikar (India)	IHL1	446,802,742	99.86%	21.9
	IHL2	436,874,056	99.67%	20.5
	IHL3	385,803,302	99.87%	18.5
	IHL4	434,986,280	99.52%	20.4
	IHL5	418,039,922	99.82%	20.2
Kangayam (India)	IKA1	433,903,903	99.86%	22.5
	IKA2	393,250,124	99.67%	20.2
	IKA3	397,852,521	99.87%	20.4
	IKA4	395,321,367	99.52%	20.4
	IKA5	463,612,943	99.82%	24.4
Kdarm Red (Cambodia)	KDR1	380,089,348	99.94%	20.3
	KDR2	421,341,141	99.93%	21.8
	KDR3	442,710,443	99.69%	24.9
	KDR4	439,812,679	99.94%	22.9
	KDR5	346,874,615	99.93%	17.9
White Cattle (Sri Lanka)	LWC1	502,173,090	99.57%	27.0
	LWC2	419,534,182	99.65%	22.7
	LWC3	398,183,637	99.93%	20.7
	LWC4	496,578,376	99.87%	25.4
	LWC5	459,735,216	99.70%	24.9
Pyar Zein (Myanmar)	YPZ1	392,433,222	99.94%	20.2
	YPZ2	426,388,364	99.92%	22.0
	YPZ3	413,520,511	99.63%	21.1
	YPZ4	448,883,061	99.93%	23.3
	YPZ5	426,713,619	99.94%	22.1
Shwe Ni (Myanmar)	YSN1	422,575,653	99.67%	21.8
	YSN2	407,604,203	99.93%	21.1
	YSN3	417,338,676	99.94%	21.4
	YSN4	407,229,133	99.69%	20.9
	YSN5	396,198,340	99.64%	20.3
Overall Mean ± SE	–	426,141,071 ± 2,019,830	99.71 ± 0.18%	21.63 ± 0.36

### 3.1. Number, distribution and genomic landscape of identified CNVRs

**Fig 1:**
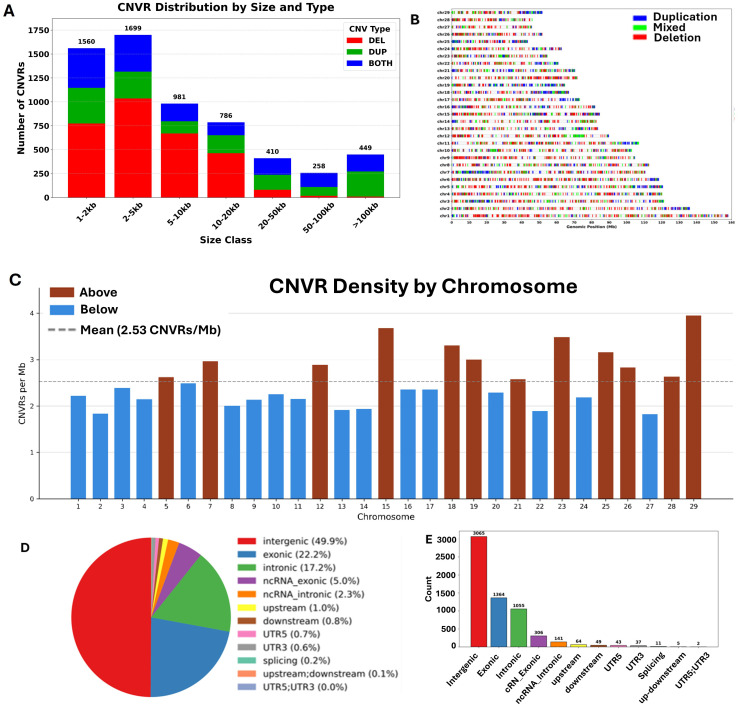
Genomic distribution of Copy Number Variation Regions (CNVRs) (a) the count and length categories of detected CNVRs (b) The autosomal distribution of CNVRs across all breeds (c) CNVR counts by chromosome length reported as CNVR density (number of CNVRs per Mb) (d) CNVRs annotations with various genomic features (e) count distribution of annotated CNVRs.

Genome wide CNV detection using CNVnator identified an average of 4627 CNVs per animal (min: 3681, max: 9254, median: 4245). After post-processing, the fraction of mapped reads with zero quality (q0 < 0.5), size >1kb and p-value <0.01 based on t-test statistics (e-val1 generated by CNVnator, a statistical t-test significance of the read depth difference between the candidate CNV region and the genome-wide background read depth distribution), we retained 7705 autosomal CNVRs. Similarly quality control filtering of CNVcaller output using the silhouette score of > 0.50 retained a total 5640 CNVRs. A sensitivity analysis was performed to assess the effect of different overlap thresholds on CNVR detection while merging the datasets (S3 File). The total number of detected CNVRs varied only modestly across thresholds, ranging from 5,992 at 30% overlap to 6,536 at 90% overlap. Hence, the 50% reciprocal overlap threshold was retained for the subsequent analysis as it provided a balance between over-merging distinct CNV events at lower thresholds and excessive fragmentation of CNVRs at higher thresholds. After merging both datasets using, a > 50% reciprocal overlap between regions, a total of 6143 high-confidence CNVRs were identified, of which 3048 were deletions, 1480 were duplications and 1615 were mixed events (both). The highest number of CNVRs was observed in Hallikar cattle breed (n = 5005) while the lowest (n = 4607) was observed in Kangayam cattle (Table 3). The total length of detected CNVRs across breeds ranged from 114.99 Mb to 163.27 Mb with average coverage of 6.23% of autosomal genome per breed, consistent with previous cattle CNV reports that ranged from 4–12% [26,27,53]. The overall mean length of identified CNVRs was 27 kb and the overall median length was 42 kb. CNVR sizes showed a right-skewed, indicating the presence of both small and moderately large CNVR events. The distribution of CNVRs based on size (Fig 1a) shows a greater number of CNVRs (n = 3259) to be under 5 kb. The distribution of CNVRs across autosomes (Fig 1b) showed chromosome 1 had the highest number of CNVRs (n = 350) covering a length of 4.7 Mb and the lowest number of CNVRs were found in chromosome 27 (n = 83) covering a length of 2.4 Mb. After normalizing by chromosome length, CNVR density ranged from 1.82 CNVRs per Mb (chromosome 27) to 3.95 CNVRs per Mb (chr 29), and a mean CNVR density revealing that smaller autosomes harboured proportionally more CNVRs than the longer ones (Fig 1c). The results of annotation analysis (Fig 1d,e) revealed 49.9% of identified CNVRs were in intergenic regions, 22.2% in the exonic regions, and 17.2% in intronic regions.

**Table 3:** Details of Copy Number Variation regions (CNVRs) identified in draught cattle breeds.

Breed	Samples	CNVRs	Total length	Median length	DUP	DEL	Mixed
Bargur	5	4802	127,973,498	4199.0	1336	2947	519
Deoni	5	4692	120,732,008	4199.0	1249	2964	479
Hallikar	5	5005	163,271,395	4599.0	1638	2899	468
Kangayam	5	4607	115,964,793	4099.0	1228	2937	442
Kdarm Red	5	4755	117,829,545	3999.0	1263	2964	528
White Cattle	5	4707	114,987,393	4299.0	1194	3031	482
Pyar Zein	5	4735	118,022,065	4199.0	1217	2992	526
Shwe Ni	5	4660	118,606,940	4199.0	1239	2904	517

### 3.2. Population differentiation based on CNVRs

**Fig 2:**
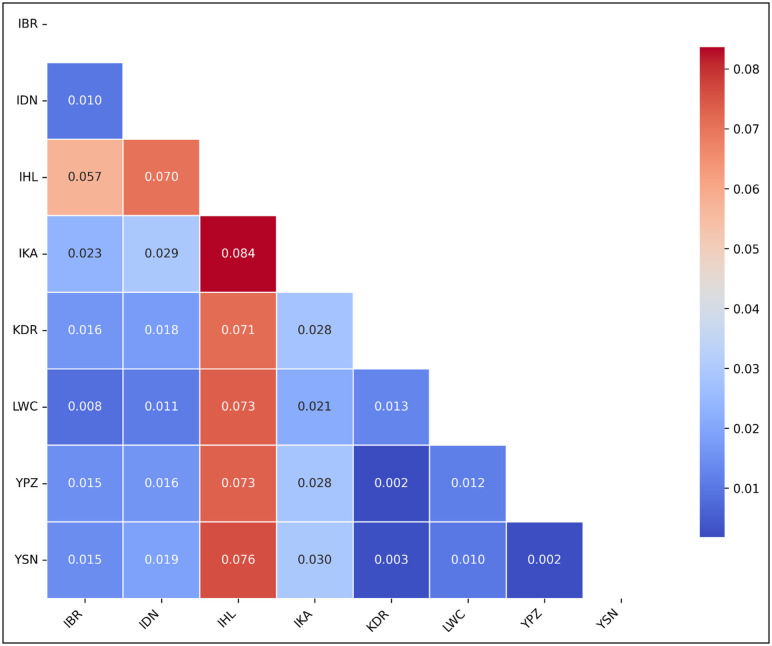
Mean pairwise V_ST_ estimates among cattle breeds based on autosomal CNVRs.

Population differentiation among breeds was evaluated using the V_ST_ statistic derived from autosomal CNVRs (Fig 2). Pairwise V_ST_ values ranged from 0.002 to 0.084, reflecting generally low to moderate levels of population differentiation (Fig 2). The Hallikar (IHL) breed from India showed relatively high overall differentiation from other investigated breeds, with a mean pairwise V_ST_ of 0.072 across all comparisons. Among all the breed-pair combinations, the largest divergence was observed between Hallikar and Kangayam (IKA) (V_ST_ = 0.084), indicating notable divergence in copy number variation between these two indigenous South Indian draught type breeds. It is also noteworthy to mention that Kangayam exhibited relatively low levels of differentiation, with V_ST_ values ranging from 0.021 to 0.030 across different pairwise comparisons. Conversely, the Southeast Asian breeds, Kdarm Red (KDR) from Cambodia and Shwe Ni (YSN) and Pyar Zein (YPZ) breeds from Myanmar showed lowest differentiation (V_ST_ = 0.002 to 0.003), suggesting CNVR similarity likely arising from shared ancestry or continuous gene flow. Most other breed pairs exhibited low differentiation (V_ST_ = 0.008 to 0.012), indicating broadly conserved CNVR patterns among them. Overall, the CNV-based population structure was modest, reflecting limited differentiation in CNVR profiles among South Asian draught cattle populations.

### 3.3. Breed-specific CNVRs

**Fig 3:**
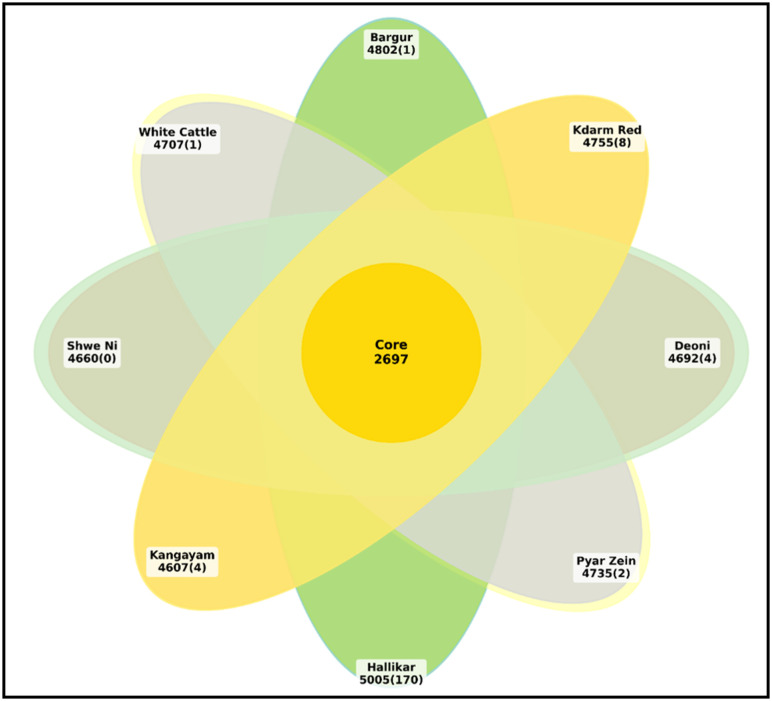
Flower plot diagram showing shared and breed-specific copy number variation regions (CNVRs) across draught cattle breeds of South Asia.

The breeds analysed in the present study are predominantly draught type cattle and hence CNVRs detected across all 40 individuals were filtered to identify both breed-specific and shared variants. A total of 6143 CNVRs were identified across the autosomes, of which 2697 (43.9%) were shared among all the eight breeds representing the core CNVR set (Fig 3). The high proportion of shared CNVRs indicates significant genomic overlap and a likely history of admixture or common ancestral origin among these indigenous populations. Among the core CNVRs, 1438 were deletions, 654 were duplications, and 605 were mixed events. Breed-specific CNVRs accounted for a small fraction of the total variation, with 190 CNVRs uniquely detected in individual breeds (Fig 3). Among these, Hallikar had the highest number of breed-specific CNVRs (n = 170). This enrichment may reflect differences in breed history or genomic diversity, however, it may also be also influenced by factors such as sample representation or detection sensitivity and therefore should be interpreted with caution. In contrast, the remaining breeds exhibited very few unique CNVRs: Kdarm Red (n = 8), Deoni (n = 4), Kangayam (n = 4), Pyar Zein (n = 2), Bargur (n = 1), White Cattle (n = 1), and Shwe Ni (n = 0). The relatively low number of unique CNVRs suggests limited detectable divergence in CNVR profiles among the zebu populations. However, given the limitations of read depth-based CNV detection, the absence of CNVRs in some breeds should not be interpreted as true biological absence, and the observed pattern may reflect both their shared utility as draught animals under similar agro-ecological conditions and methodological constraints.

### 3.4. Functional annotation of CNVR overlapping genes/QTLs

**Fig 4:**
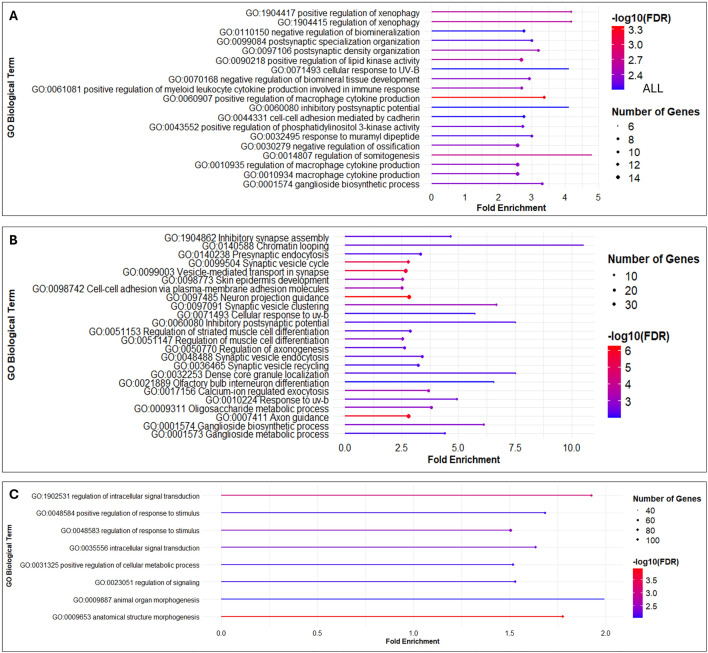
Top GO biological processes significantly enriched (p < 0.05) for (A) all CNVRs (B) core CNVRs shared across all breeds and (C) Hallikar-specific CNVRs.

**Fig 5:**
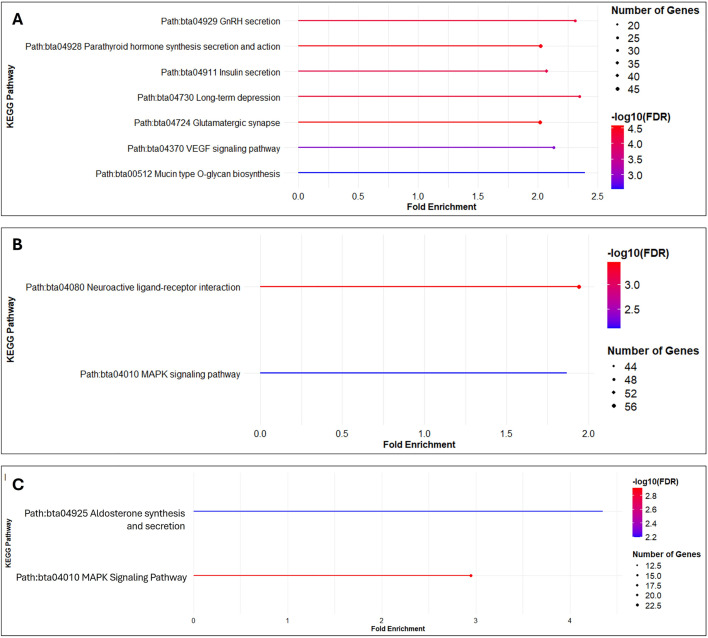
Top KEGG pathways significantly enriched (p < 0.05) for (A) all CNVRs (B) core CNVRs shared across all breeds (C) Hallikar-specific CNVRs.

Functional annotation of all 6143 CNVRs (detected across all eight breeds) using *Bos indicus* reference genome resulted in identification of 4868 genes overlapping the structural variants, where a gene was considered to overlap a CNVR if any portion of its genomic coordinates intersected with the CNVR boundaries. The core set of 2697 shared CNVRs overlapped with 2381 genes while the 170 Hallikar specific CNVRs annotated to 618 genes. Functional analysis for detection of enriched Gene Ontology Biological Processes (GOBP) and KEGG pathways using 4868 genes spanning across all the CNVRs were conducted, the results of which are summarized in Figs 4, 5 and (S4 File).

For the complete dataset comprising 4,868 genes overlapping 6,143 CNVRs, enrichment analysis identified 19 GOBP terms with fold enrichment greater than 2.5 (Fig 4A). The enriched categories included processes related to immune regulation, cytokine production, lipid metabolism, cellular stress response, cell adhesion, and developmental regulation. The representative GOBP terms included positive regulation of macrophage cytokine production, regulation of somitogenesis, positive regulation of xenophagy, postsynaptic density organization, cell–cell adhesion mediated by cadherin, and response to UV-B radiation. The 2,381 genes overlapping the core CNVR set revealed 24 significantly enriched GOBP terms (Fig 4B), including categories associated with neuronal development, synaptic organization, vesicle-mediated transport, and signal transduction. Enriched terms included neuron projection guidance, axon guidance, synaptic vesicle cycling, and calcium-ion regulated exocytosis. In the Hallikar breed-specific CNVRs, eight GOBP terms were significantly enriched (Fig 4C), mainly involving morphogenesis, intracellular signalling, and metabolic regulation. The prominent GOBP terms included anatomical structure morphogenesis, regulation of intracellular signal transduction, and positive regulation of cellular metabolic process.

KEGG pathway enrichment analysis identified several pathways associated with signalling, neuroendocrine regulation, and metabolism. In the complete dataset, seven pathways were significantly enriched (Fig 5A), including glutamatergic synapse, parathyroid hormone synthesis and secretion, long-term depression, gonadotropin-releasing hormone (GnRH) secretion, insulin secretion, VEGF signalling, and mucin-type O-glycan biosynthesis. The core CNVR set showed enrichment of two pathways, namely neuroactive ligand–receptor interaction and MAPK signalling (Fig 5B). Similarly, Hallikar-specific CNVRs showed enrichment for MAPK signalling and aldosterone synthesis and secretion pathways (Fig 5C).

To examine the potential relationship between CNVRs and economically important traits, CNVR coordinates from the complete, core, and Hallikar-specific datasets were intersected with cattle QTLs available in the Animal QTLdb [66]. With the complete set of 6,143 CNVRs, 65 unique QTLs were identified (Table 4), including QTLs related to carcass traits (33 QTLs), fertility and reproduction [13], growth [6], disease susceptibility and immune-related traits [4], milk mineral composition [3], and other miscellaneous traits [6]. The overlapping QTLs included those associated with antral follicle number, Anti-Müllerian hormone levels, average daily gain, carcass weight, somatic cell score, subcutaneous fat thickness, muscularity, and mineral composition traits. The core set of 2,697 shared CNVRs overlapped with 40 unique QTLs (Table 4) while the 170 Hallikar-specific CNVRs overlapped with nine unique QTLs, comprising five carcass-related QTLs, three QTLs associated with mineral composition traits, and one growth-related QTL. Overall, the QTL overlap analysis identified CNVRs located within genomic intervals previously reported to be associated with economically important traits in cattle.

**Table 4:** Summary of QTLs overlapping with ALL, core (shared) and Hallikar-specific CNVRs.

Quantitative trait Category	Quantitative trait	No. QTLs					
		ALL CNVRs	Total	Core CNVRs	Total	Hallikar-specific CNVRs	Total
Carcass traits	Carcass weight	1	33	–	21	–	5
	Finishing precocity	1		–		–	
	Juiciness	1		1		–	
	Longissimus muscle area	6		4		2	
	Marbling score	3		3		–	
	Meat color	2		1		–	
	Meat firmness	1		1		–	
	Tenderness score	2		2		–	
	Subcutaneous fat thickness	16		9		3	
Growth	Average daily gain	1	6	–	3	–	1
	Body weight	1		–		–	
	Conformation score	1		1		–	
	Muscularity	3		2		1	
Fertility & Reproductive traits	Anti-MÃllerian hormone level	5	13	3	8	–	
	Antral follicle number	8		5		–	
Disease susceptibility/Resistance	M. paratuberculosis susceptibility	3	4	3	4	–	
	Somatic cell score	1		1		–	
Milk composition (Minerals)	Milk calcium content	1	3	–		1	3
	Milk magnesium content	1		–		1	
	Milk phosphorus content	1		–		1	
Others	Blood calcium level	2	6	1	4	–	
	Intestinal atresia	2		1		–	
	Mean corpuscular hemoglobin concentration	1		1		–	
	Ketosis	1		1		–	
Total		65	65	40	40	9	9

## 4. Discussion

Genomic diversity in zebu cattle of South Asia has been studied extensively using single nucleotide polymorphisms (SNPs) [35,67,68], microsatellite and mitochondrial DNA markers [39,69]. However, comprehensive information on the diversity and distribution of structural variations in Asian zebu cattle genome remains limited. Most of the CNV analysis in zebu breeds relied on high quality taurine reference genome assemblies such as ARS-UCD1.2 [70], UMD3.1 [71], Btau_4 [72], and UOA_Angus_1 [73]. The recent availability of a *Bos indicus* reference genome NIAB_ARS_BosIndicus_Tharparkar_1.0 (GCF_029378745.1) provided an improved framework for CNV detection by enhancing breakpoint resolution and reducing reference bias. Vani et al [53] investigated CNVs using *Bos indicus* Nellore reference genome (GCF_ 000247795.1_Bos_indicus_1.0) [74] in 15 pooled samples belonging to five zebu draught breeds. In the present study we expanded the dataset to include 40 individuals from eight draught breeds across India, Sri Lanka, Myanmar and Cambodia, thereby broadening the geographic and genetic representation of South and Southeast Asian Zebu populations.

CNVRs were identified with high reliability with an average sequencing depth of 21.2 × , which is higher than 8–10 × reported for earlier South Asian cattle studies [53,23,49,75]. The number of CNVRs detected in the present study is broadly comparable to previous reports across diverse cattle populations, including Asian and Indian draught breeds (11,065 CNVRs) [53], indigenous Chinese cattle populations (ranging from 1,651–9,349 CNVRs) [76], Hainan cattle (5,458 CNVRs) [77], Pinan and Nanyang cattle (9,631 CNVRs) [78], Nellore cattle (1,884 CNVRs) [54], Tharparkar cattle (447 CNVRs) [52], and Ethiopian indigenous cattle breeds (3,893 CNVRs) [27]. The CNV-based genomic architecture inferred in this study is consistent with earlier investigations, particularly with respect to autosomal CNV coverage which accounted for approximately 6.3% of the genome. This estimate falls within the range reported for most cattle populations [8,76,78,79], although it is lower than the substantially higher CNV coverage (11–17%) reported in draught cattle populations of Tamil Nadu, India [53]. Furthermore, a higher proportion of deletions relative to duplications was observed, a pattern that has been consistently reported in CNV studies across cattle and other mammalian species [12,27,31,80].

Population differentiation assessed using CNVR derived V_ST_ values revealed low to moderate but significant structural genomic divergence among the investigated draught cattle populations. The observed range of pairwise V_ST_ values (0.002–0.084) indicated a relatively low magnitude of differentiation, consistent with the shared zebu ancestry and common evolutionary pressures associated with draught utility and adaptation to tropical agro-ecological environments. While most breeds share broadly conserved CNVR patterns, certain breeds showed relatively higher differentiation. Specifically, Hallikar cattle from southern India displayed the highest observed value for CNV based differentiation, as evidenced by the highest mean pairwise V_ST_ values across comparisons. The significant divergence observed between Hallikar and Kangayam cattle, despite their geographic proximity, may indicate distinct selective breeding practices resulting in differential accumulation of structural variants. This divergence could reflect long-term selection for functional traits such as endurance, gait efficiency, or workload specialization, which are known to vary subtly among traditional draught breeds. In contrast, the Southeast Asian breeds, Kdarm Red from Cambodia and Shwe Ni and Pyar Zein from Myanmar showed low levels of CNV based differentiation, indicative of highly similar CNVR profiles. This genomic homogeneity likely reflects historical relatedness or continuous gene flow among them, potentially facilitated by transboundary livestock movement and overlapping production systems. Similarly, most Indian indigenous breeds and Sri Lankan cattle exhibited low pairwise V_ST_ values, pointing to broadly conserved structural genomic variations among them. The limited differentiation observed among these populations suggests that adaptive divergence may be concentrated in a small subset of CNVRs rather than being genome-wide, highlighting the role of specific structural variants in mediating local adaptation. However, it is important to mention that CNV detection using read depth-based approaches is influenced by the choice of filtering and detection parameters. These settings may preferentially retain high confidence CNVRs while reducing sensitivity to rare or low frequency variants. As a result, the observed distribution of differentiation and breed specific CNVRs may be partially affected by methodological constraints in addition to underlying biological variation.

Enrichment analysis identified GO terms that were also reported in other Asian cattle from China, and India [53,76]. CNVR overlapping genes involved in immune regulation, such as cytokine and chemokine signalling (for example *CXCL2*, *CCL19*, and other interleukin-related genes), likely affect macrophage activation and host defence pathways. These cytokines and chemokines are central to pro-inflammatory responses and leukocyte recruitment, shaping innate immune response to pathogens common in tropical cattle production environments. Similar enrichment of immune related genes has been reported in previous cattle CNV studies, indicating potential association of structural variants with disease resistance and environmental resilience [27,81,82]. Further, CNVRs intersecting genes linked to cellular stress responses and tissue integrity may indicate functional impact. For example, variants in genes that mediate cellular response to damage or pathogen exposure may influence the threshold and extent of stress responses, potentially affecting heat tolerance or oxidative stress regulation [77,83]. Within the core CNVRs shared across breeds, neuronal and synaptic processes were strongly overrepresented. Enrichment of neuron projection guidance, synaptic vesicle cycling, and calcium-dependent exocytosis points to the involvement of genes such as *RAB3A* and related vesicle-trafficking regulators, which are critical for controlled neurotransmitter release and effective neuromuscular signal transmission. These processes indicate coordinated movement and sustained muscle activity, suggesting a potential role of structural variants in behavioural stability, stamina, and work efficiency. Calcium-dependent exocytosis processes are conserved mechanisms mediating synaptic vesicle release and are critical for efficient nerve-to-muscle signalling that underlies coordinated movement and stamina [84].

KEGG pathway enrichment analysis revealed that genes overlapping CNVRs are distributed across signalling pathways that regulate neuroendocrine control, reproduction, stress adaptation, growth, and metabolic homeostasis. Among the most significantly enriched pathways were glutamatergic synapse and long-term depression, indicating that structural variation affects genes central to neuronal signalling. These pathways included genes such as *GNAI1*, *GNAI2*, *GNB2*, *GNG2*, *ADCY7*, *ITPR2*, and *RYR1*, which regulate neurotransmitter-mediated calcium signalling and downstream second-messenger responses [85,86]. Glutamatergic and calcium-dependent signalling is essential not only for central nervous system function but also for neuromuscular coordination, behavioural responses, and stress perception [85,87,88]. The enrichment of the GnRH secretion pathway highlights the role of CNVR-associated genes in reproductive endocrinology. This pathway comprised key regulators such as *KISS1*, *KISS1R*, *PLCB1*, *PLCB2*, *AKT1*, *ITPR1*, *ITPR2*, and multiple voltage-gated calcium and potassium channel genes (*CACNA1* and *KCNN* families). These genes collectively regulate pulsatile GnRH release and pituitary gonadotropin secretion, thereby influencing ovulation rate, oestrous cyclicity, and fertility. Structural variation affecting these components may contribute to differences in reproductive efficiency and adaptability under environmental stress [89]. Pathways related to hormonal and stress regulation, including parathyroid hormone synthesis and secretion and aldosterone synthesis and secretion, were also significantly enriched. Genes such as *GNA11*, *ADCY7*, *BCL2*, and *MEF2D* link calcium homeostasis, endocrine signalling, and cellular survival. These pathways play important roles in mineral balance, muscle contraction, and physiological adaptation to nutritional and thermal challenges [90]. Growth- and metabolism-associated pathways, including MAPK signalling, insulin signalling, Rap1 signalling, VEGF signalling, and endocytosis, were also consistently over-represented. Core genes such as *MAP2K1*, *MAP2K5*, *MAPK7*, *PRKCA*, *AKT1*, *HRAS*, and *CDC42* regulate cell proliferation, angiogenesis, glucose uptake, and nutrient partitioning between muscle and adipose tissues. Variation in these pathways has been associated with growth rate, feed efficiency, muscle development, and fat deposition in cattle [91,92]. Overall, the KEGG pathway enrichment profile indicated intersection of CNVRs with highly integrated neuroendocrine, metabolic, and stress-responsive pathways, suggesting the potential influence of structural variation on coordinated regulation of reproduction, growth, and resilience in indigenous cattle populations raised under challenging production environments. Although these functional enrichments provided an indication of the biological functions represented within the CNVR-overlapping genes; it is important to exercise caution while interpreting their association with specific adaptive/productive characteristics in the studied cattle populations.

The overlap of CNVRs with carcass-related QTLs [83,93,94] indicates that copy number changes are located in genomic regions influencing muscle development, fat deposition, and body conformation. The detection of CNVRs in QTL regions associated with fertility and reproduction traits [17,29] suggests that structural variation may influence reproductive efficiency in low-input production systems, where animals must allocate limited resources between basic physiologic/metabolic needs, work and reproduction. Similarly, the overlap of core set of shared CNVRs with QTLs linked to disease susceptibility and immune responsiveness points to a role for copy number variation in tolerance to infection challenges [95,96].

## 5. Conclusion

The present study provides a comprehensive genome-wide assessment of copy number variation regions in South Asian indigenous draught cattle using whole-genome sequencing data. A large number of CNVRs were identified across eight breeds, with a substantial proportion shared among populations while a smaller number of breed-specific CNVRs were detected. Functional enrichment analyses showed that genes overlapping CNVRs were predominantly involved in immune regulation, metabolic control, neuroendocrine signalling, and cellular responses to stress, indicating biological processes relevant to adaptation, work capacity, and general fitness. Mapping of CNVRs to known quantitative trait loci further supported associations with carcass characteristics, growth, fertility, and reproduction traits. Overall, the findings expand current knowledge of structural genomic diversity in South Asian draught cattle and provide baseline genomic information that can support future studies on their adaptation, breeding, management and conservation.

## Supporting information

S1 File Declaration on Inclusivity in Global Research. (DOCX)

S2 File List of background gene set used for enrichment analysis using ShinyGo. (TXT)

S3 File Sensitivity analysis to assess the effect of different overlap thresholds on CNVR detection. (DOCX)

S4 File List of enriched GO processes and KEGG pathways involving genes overlapping with ALL, core (shared) and Hallikar-specific CNVRs. (XLSX)
