# Supplementary material for: Genome-wide characterization of copy number variants and their functional relevance in indigenous draught cattle of South Asia
Source: PLoS One. 2026 Jul 13;21(7):e0353468. doi: 10.1371/journal.pone.0353468 (PMC13362094; doi:10.1371/journal.pone.0353468)
Supplement: S3 File — (DOCX) [file pone.0353468.s003.docx]

**Supporting Information S3 File. Sensitivity analysis to assess the effect of different overlap thresholds on CNVR detection**

A sensitivity analysis was performed using reciprocal overlap thresholds of 30%, 50%, 70%, and 90% for CNVR construction. The total number of detected CNVRs varied only modestly across thresholds, ranging from 5,992 at 30% overlap to 6,536 at 90% overlap. Relative to the 50% threshold used in the primary analysis, CNVR counts differed by −2.46%, +2.02%, and +6.40% at 30%, 70%, and 90%, respectively. Similar trends were observed for total genomic coverage and CNVR classification categories, indicating that the overall CNVR landscape was robust to the choice of overlap threshold.

The 50% reciprocal overlap threshold was retained for the main analysis because it provides a balance between over-merging distinct CNV events at lower thresholds and excessive fragmentation of CNVRs at higher thresholds. Furthermore, a 50% reciprocal overlap criterion has been widely adopted in previous CNV studies (Keel et al. 2016; Letaief et al. 2017; Butty et al. 2020; Buggiotti et al. 2022; Braga et al. 2023), facilitating comparison with published datasets. These results demonstrate that the conclusions of the study are not sensitive to the overlap threshold selected for CNVR construction.

| Threshold | CNVR_count | Total_bp | Pure_deletion | Pure_duplication | Mixed |
| --- | --- | --- | --- | --- | --- |
| 0.3 | 5,992 | 167,110,108 | 3,025 | 1,410 | 1,557 |
| 0.5 | 6,143 | 170,730,457 | 3,048 | 1,480 | 1,615 |
| 0.7 | 6,267 | 173,818,533 | 3,082 | 1,534 | 1,651 |
| 0.9 | 6,536 | 180,020,764 | 3,195 | 1,622 | 1,719 |

The above description and results of the sensitivity analysis is now included as Supporting Information File S4 in the revised version of the manuscript.

**References:**

1. Keel BN, Lindholm-Perry AK and Snelling WM (2016) Evolutionary and Functional Features of Copy Number Variation in the Cattle Genome. Front. Genet. 7:207. doi: 10.3389/fgene.2016.00207
2. Letaief, R., Rebours, E., Grohs, C. *et al.* Identification of copy number variation in French dairy and beef breeds using next-generation sequencing. *Genet Sel Evol* **49**, 77 (2017). <https://doi.org/10.1186/s12711-017-0352-z>
3. Butty, A.M., Chud, T.C.S., Miglior, F. *et al.* High confidence copy number variants identified in Holstein dairy cattle from whole genome sequence and genotype array data. *Sci Rep* **10**, 8044 (2020). <https://doi.org/10.1038/s41598-020-64680-3>
4. Buggiotti, L., Yudin, N. S., & Larkin, D. M. (2022). Copy Number Variants in Two Northernmost Cattle Breeds Are Related to Their Adaptive Phenotypes. Genes, 13(9), 1595. <https://doi.org/10.3390/genes13091595>
5. Braga, L. G., S. Chud, T. C., Watanabe, R. N., Savegnago, R. P., Sena, T. M., Machado, M. A., Panetto, C., & Munari, D. P. (2023). Identification of copy number variations in the genome of Dairy Gir cattle. *PLOS ONE*, *18*(4), e0284085. <https://doi.org/10.1371/journal.pone.0284085>
