# Supplementary material for: Genome-wide characterization of copy number variants and their functional relevance in indigenous draught cattle of South Asia
Source: PLoS One. 2026 Jul 13;21(7):e0353468. doi: 10.1371/journal.pone.0353468 (PMC13362094; doi:10.1371/journal.pone.0353468)
Supplement: Attachment — Submitted filename: 1.Mavunga_Response_To_Reviewers.docx [file pone.0353468.s006.docx]

**RESPONSE TO REVIEWERS’ COMMENTS**

**EDITOR’S COMMENTS**

Thank you for submitting your manuscript to PLOS ONE. After careful consideration, we feel that it has merit but does not fully meet PLOS ONE’s publication criteria as it currently stands. Therefore, we invite you to submit a revised version of the manuscript that addresses the points raised during the review process.

**Authors’ Response:** Thanks for the comment. The manuscript is now revised as per the comments of both the reviewers

**REVIEWER 1 COMMENTS**

The manuscript's topic is of interest. The study provides valuable insights into the genome-wide characterization of copy number variation regions (CNVRs) in eight indigenous Asian zebu cattle, traditionally used for ploughing, wet-field agriculture, and carting. Several genes overlapping these CNVRs were associated with immune function, metabolism, and adaptation, and some regions corresponded to QTLs linked to carcass, fertility, reproduction, and growth traits. The findings provide important genomic resources for understanding structural variation and support cattle breeding and conservation programs. Overall, the manuscript is well-structured, and the findings are supported by vigorous data analysis.

**Authors’ Response:** We thank the reviewer for their positive evaluation of the manuscript and for their overall comment.

**Reviewer 1 Comment:** Introduction lines 100-128, the examples of breeds are lengthy. Consider shortening these examples to make the introduction more concise.

**Authors’ Response:** Thank you for the comment, we have shortened examples of breeds to allow conciseness of the introduction.

**Reviewer 1 Comment:** Lines 153–155: “eight distinct breeds was performed with five samples per breed: Kangayam, Hallikar, Bargur and Deoni from India, White Cattle from Sri Lanka, Kdarm Red from Cambodia, Pyar Sein and Shwe Ni cattle from Myanmar” could be presented in a table format (e.g., breed name, country of origin, and number of samples). This would make the information easier to read.

**Authors’ Response:** We thank the reviewer for their comment, we have included the table in the manuscript at suggested.

**REVIEWER 2 COMMENTS**

This manuscript presents a genome-wide characterization of copy number variation regions across eight indigenous South Asian zebu cattle breeds using read-depth-based approaches, followed by analyses of population differentiation, functional annotation, and QTL overlap. The topic is relevant, and the dataset has value, particularly because structural variation remains under characterized in many indigenous cattle populations. The use of two CNV callers, genome-wide cataloguing, and downstream functional analyses provides a potentially useful baseline resource.

Overall, I find the study technically sound, with an acceptable methodology but limited biological depth and consistent overinterpretation of results. The study is suitable for publication after major revision. My main concerns center on methodological justification, reporting clarity, and overinterpretation of the biological significance of the results. In particular, the CNV detection framework is broadly reasonable but requires additional explanation in several places; the population differentiation results are modest and should be interpreted more cautiously; the Hallikar-specific findings need stronger support; and the enrichment/QTL sections currently read as overly descriptive and more speculative than the data warrant.

**Author Response:** We thank the reviewer for their positive evaluation of the manuscript and for their overall comment. The main concerns/suggestions of the reviewer are addressed in the revised manuscript.

**INTRODUCTION**

**Reviewer Comment:** At lines 68-78, the discussion of CNVs, their size range, mechanisms of origin, and effects on gene expression should be more fully referenced.

**Author Response:** Thank you for the comment, we have adjusted the section and included the below references to support the statements**.**

Hastings, P., Lupski, J., Rosenberg, S. *et al.* Mechanisms of change in gene copy number. *Nat Rev Genet* **10**, 551–564 (2009). <https://doi.org/10.1038/nrg2593>

Louise Harewood, Peter Fraser, The impact of chromosomal rearrangements on regulation of gene expression, *Human Molecular Genetics*, Volume 23, Issue R1, 15 September 2014, Pages R76–R82, <https://doi.org/10.1093/hmg/ddu278>

Eric R. Gamazon, Barbara E. Stranger, The impact of human copy number variation on gene expression, *Briefings in Functional Genomics*, Volume 14, Issue 5, September 2015, Pages 352–357, <https://doi.org/10.1093/bfgp/elv017>

Clop, A., Vidal, O. and Amills, M. (2012), Copy number variation in the genomes of domestic animals. Anim Genet, 43: 503-517. [https://doi.org/10.1111/j.1365 2052.2012.02317.x](https://doi.org/10.1111/j.1365%202052.2012.02317.x)

Fadista, J., Thomsen, B., Holm, LE. *et al.* Copy number variation in the bovine genome. *BMC Genomics* **11**, 284 (2010). <https://doi.org/10.1186/1471-2164-11-284>

**Reviewer Comment:** The section around lines 90-94 and 100-109 also needs supporting citations, especially where the manuscript summarizes the major computational approaches for CNV detection and their advantages.

**Author Response:** Thank you for the comment we have added the below references to support section 90-94.

Zhao M, Wang Q, Wang Q, Jia P, Zhao Z. Computational tools for copy number variation (CNV) detection using next-generation sequencing data: features and perspectives. BMC Bioinformatics. 2013 Sep 13;14(S11):S1. doi:10.1186/1471-2105-14-S11-S1

Zhang ZD, Du J, Lam H, Abyzov A, Urban AE, Snyder M, et al. Identification of genomic indels and structural variations using split reads. BMC Genomics. 2011 Dec 25;12(1):375. doi:10.1186/1471-2164-12-375

Pirooznia M, Goes FS, Zandi PP. Whole-genome CNV analysis: advances in computational approaches. Front Genet. 2015 Apr 13;06.doi:10.3389/fgene.2015.00138

Whitford W, Lehnert K, Snell RG, Jacobsen JC. Evaluation of the performance of copy number variant prediction tools for the detection of deletions from whole genome sequencing data. J Biomed Inform. 2019 Jun;94:103174. doi:10.1016/j.jbi.2019.103174

Yoon S, Xuan Z, Makarov V, Ye K, Sebat J. Sensitive and accurate detection of copy number variants using read depth of coverage. Genome Res. 2009 Sep;19(9):1586–92. doi:10.1101/gr.092981.109

**Author Response:** Thank you for the comment we have added the below references to support section 100-109. These references describe the functional uses of the breeds, their origins and characteristics.

Mavunga, T.K., Sölkner, J., Mészáros, G. *et al.* Genomic diversity and selection signatures in Asian Zebu Cattle: insights into adaptation and genetic erosion. *Sci Rep* **15**, 33346 (2025). <https://doi.org/10.1038/s41598-025-14744->z

Singh PK. Phenotypic characterization and performance evaluation of Hallikar cattle in its native tract. Indian Journal of Animal Sciences. 2008;78(2):211–4.

Kuralkar SV. Phenotypic characteristics, production and reproduction performance of Deoni cattle in its native tract. . Indian Journal of Animal Sciences,. 2014;84(1):75–7.

Hattarakihal M, Patil VM, Waghmare PG, Suranagi M, Kulkarni S, Desai AR, et al. Morphometric traits of different Deoni cattle strains. International Journal of Veterinary Sciences and Animal Husbandry. 2023 Jan 1;8(4S):151–4. doi:10.22271/veterinary.2023.v8.i4Sc.663

Lokugalappatti LGS, Wickramasinghe S, Alexander PABD, Abbas K, Hussain T, Ramasamy S, et al. Indigenous cattle of Sri Lanka: Genetic and phylogeographic relationship with Zebu of Indus Valley and South Indian origin. PLoS One. 2023 Aug 1;18(8 August). doi:10.1371/journal.pone.0282761 PubMed PMID: 37585485.

Lwin M, Mon SLY, Yamanaka H, Nagano Y, Mannen H, Faruque MO, et al. Genetic diversities and population structures of four popular Myanmar local cattle breeds. Animal Science Journal. 2018 Dec 14;89(12):1648–55. doi:10.1111/asj.13112

Namikawa T. MH, TK, NK, TY,. Coat-color variations of cattle observed in the fields of Cambodia, and withers-height and other traits in the native cattle subjected to further experimental analyses. Rep Soc Res Native Livestock. 2006; 23:31–44.

**Reviewer Comment:** The breed descriptions at lines 105-120 could also be improved. A concise breed reference may be sufficient in the main text, while more detailed breed descriptions or representative photos could be moved to the Supplementary Information if the authors wish to provide additional context.

**Author Response:** We thank the reviewer for this helpful suggestion. The breed descriptions in lines 105–120 have been revised to improve conciseness and readability. In the main text, we now provide only brief information on breed origin and key functional traits relevant to the study. More detailed descriptions have been removed to avoid redundancy and maintain focus.

**Reviewer Comment:** 132-134, The manuscript would benefit from a more focused introduction that clearly summarizes what is currently known about CNVs in Asian zebu cattle. This should include key findings from previous studies, commonly used methodologies, and any existing limitations in the literature. In addition, the authors should explicitly define the specific gap this study addresses and the new insights it aims to provide. Clarifying this will help the reader better understand the motivation and the precise contribution of the work.

**Author Response:** We thank the reviewer for this insightful suggestion. The Introduction has been revised to provide a clearer overview of copy number variations (CNVs) in Asian zebu cattle, with particular emphasis on South Asian and Southeast Asian populations. We have incorporated relevant literature describing CNV patterns in indicine cattle and outlined commonly used methodologies for CNV detection, including array-based and next-generation sequencing approaches.

In addition, we have highlighted key limitations in the existing literature, such as the underrepresentation of indigenous zebu breeds from South and Southeast Asia and challenges related to CNV detection and comparability across studies. Finally, we have clarified the specific knowledge gap addressed by this study and explicitly stated its contribution to improving the understanding of CNV diversity in South and Southeast Asian zebu cattle.

**MATERIALS AND METHODS**

**Reviewer Comment:** At lines 155–159, the manuscript should provide more explicit information about the source of the DNA samples, how genomic DNA was extracted or processed, and the sequencing design. It is also important to state whether the reads were single-end or paired-end, their read length, and the target or achieved sequencing depth per sample. These details are necessary for reproducibility and for evaluating the suitability of the data for read-depth–based CNV calling.

**Author Response:** Thanks for the comment and the opportunity to clarify our sample collection and DNA extraction strategy. All the DNA samples used in the present study were derived from the existing Genetic Repository at Animal Production and Health Laboratory, Joint FAO/IAEA Centre of Nuclear Techniques in Food and Agriculture, International Atomic Energy Agency, Vienna, Austria (Already indicated under the Ethics statement in the Methods section). The DNA available at the FAO/IAEA repository were extracted from blood samples collected by jugular venipuncture into EDTA vacutainer tubes and isolated using MasterPure DNA Purification Kit (Biozym, Illumina Inc, USA). It was ensured that the samples included in the study were collected from unrelated cattle, based on the information available in the Genetic Repository module of FAO/IAEA Genetic Laboratory Information and Data Management System (GLIDMaS). We have revised the manuscript to include the above information for better clarity. We have also added details of sequencing design, specifying that paired-end sequencing was used, along with read length. In addition, the average sequencing depth per sample, has been included in the revised version of the manuscript.

**Reviewer Comment:** The use of CNVnator and CNVcaller is a reasonable approach for CNV discovery from short-read data. However, several aspects of the methodology here still need clarification and justification. For instance, at lines 179–180, the authors should justify the use of a 100 bp bin size in CNVnator. This choice increases nominal resolution but can also increase susceptibility to local coverage noise. It would also be helpful to know whether alternative bin sizes were considered and why 100 bp was ultimately selected.

**Author Response:** Thank you for this valuable comment. We agree that precision in breakpoint location is a function of bin size in CNVnator. As per the developers of CNVnator (Abyzov et al. 2011), the choice of bin size is a function of coverage, read length, and data quality. Given the data quality and read length remaining same, the optimal bin size (and thus breakpoint resolution accuracy) scales inversely with the coverage. Hence, Abyzov et al (2011) recommended a bin size of ∼100-bp for sequence data with 20–30× coverage, ∼500-bp bins for 4–6× coverage, and ∼30-bp bins for ∼100× coverage. Hence, in the present study we chose a bin size of 100bp since the mean coverage of our sequence data was 21.63 ± 0.36.

**Reviewer Comment:** At lines 195–197, the generation of custom duplicated window files and a custom reference setup for CNVcaller introduces reproducibility concerns. The authors should describe more clearly how this resource was generated and validated and ideally make the pipeline or script publicly available.

**Author response:** Thank you for this comment. We like to clarify that the duplicated window files and reference database were generated strictly following the official CNVcaller pipeline provided by the developers (<https://github.com/JiangYuLab/CNVcaller>). Their inbuilt database is made from the taurine reference genome, and they provide steps to guide one to make their own custom database for subspecies like indicine. We have revised the Methods section to explicitly describe the key steps involved using the provided scripts. To further enhance reproducibility, we have provided exact commands used in this study under the Methods section in the revised manuscript enabling full replication of the workflow.

**Reviewer Comment:** At lines 201–205, the CNVcaller filters -f 0.1 and -h 3 may bias the analysis toward common CNVs while excluding low-frequency or truly breed-specific events. This is particularly relevant because the manuscript later interprets the biological significance of shared versus breed-specific CNVRs. The authors should discuss how these thresholds may influence the observed CNVR spectrum and whether they reduce sensitivity to rare variants.

**Author response:** We thank the reviewer for this comment. We agree that the choice of filtering thresholds in CNVcaller can influence the resulting CNVRs detected, and the balance between sensitivity and specificity. In our study we applied filtering thresholds as recommended in the CNVcaller framework (<https://github.com/JiangYuLab/CNVcaller>) to reduce false-positive CNV calls arising from stochastic read-depth variation and low-support signals, particularly given the ~20x sequencing depth used in this study. These parameters are commonly used to retain CNVs supported by a minimum proportion of individuals while ensuring robustness of detected variants (Deng TX, Ma XY, Duan A, Lu XR, Abdel-Shafy H. Genome-wide copy number variant analysis reveals candidate genes associated with milk production traits in water buffalo (Bubalus bubalis). J Dairy Sci. 2024 Sep;107(9):7022-7037. doi: 10.3168/jds.2023-24614) (Liu Y, Mu Y, Wang W, Ahmed Z, Wei X, Lei C and Ma Z (2023) Analysis of genomic copy number variations through whole-genome scan in Chinese Qaidam cattle. Front. Vet. Sci. 10:1148070. doi: 10.3389/fvets.2023.1148070). We acknowledge that these filtering criteria may reduce sensitivity to rare or low-frequency CNVs, including potentially breed-specific variants present at low prevalence. As a result, the CNVRs reported here represent a conservative set of high-confidence variants, and the interpretation of shred versus breed-specific CNVRs is focused on variants that pass these stringent filters. This limitation and its implications for the bias provided by the filters have been discussed in the revised manuscript.

**Reviewer Comment:** At line 204, the use of a 50% reciprocal overlap criterion for merging CNVs into CNVRs should be explicitly justified. This is a common heuristic, but it remains an arbitrary threshold that can affect the number and size of CNVRs. If possible, a brief sensitivity check or rationale would strengthen the methodological rigor.

**Author response:** Thank you for this comment. We agree that the choice of reciprocal overlap can influence CNVR definition and downstream interpretation. In addition to the 50% reciprocal overlap criterion used in this study, we evaluated alternative thresholds (including more stringent and more relaxed overlap criteria) to assess the stability of CNVR formation. While these alternative settings influence the total number and fragmentation of CNVRs to some extent, the overall distribution patterns and major CNVR signals remained consistent. The 50% threshold was therefore selected as it provides a balanced compromise between the merging closely related CNVs and avoiding over-consolidation of distinct events. This cutoff is also widely used in CNV studies and facilitates comparability with previously published work.

**RESULTS**

**Reviewer Comment:** At line 236, the reported sequencing metrics, including mean coverage around 21.2× and mapping rate around 99%, indicate good overall data quality and support the use of read-depth–based CNV detection in this dataset. However, the manuscript’s wording that this enables “accurate CNV detection” is somewhat too strong given the known limitations of read-depth methods, especially in repetitive or low-mappability regions. A more cautious phrasing would be appropriate.

**Author response:** Thank you for the comment, we agree that our statement may be too strong given the inherent limitations of read depth-based approaches. We have revised the wording in the manuscript to appropriately reflect these methodological considerations.

**Reviewer Comment:** At lines 244–245, the statement that CNVs were retained based on “p-value < 0.01 based on t-test statistics” requires more detail. CNVnator reports multiple p-value-related metrics, and it is currently unclear which statistic the authors used. The manuscript should clarify which CNVnator p-value output was applied, how that p-value is defined within CNVnator, and whether any multiple-testing considerations were taken into account. The wording should also make clear that this is part of CNVnator’s internal model rather than a separate test performed independently by the authors.

**Author response:** We thank the reviewer for this comment. We clarify that the p-value referred to in the manuscript corresponds to e-val1, which is based on a t-test statistical significance of the read depth difference between the candidate CNV region and the genome-wide background read depth distribution. Thus, this p-value is not derived from an independent statistical test performed by authors but is part of CNVnator’s built-in model for CNV calling. We have revised the text in the manuscript to reflect this notion.

**Reviewer Comment:** At lines 251–259, the manuscript reports that CNVRs cover approximately 6.23% of the autosomal genome. This should be contextualized against previous cattle CNV studies. The authors should also clarify whether repetitive regions or segmental duplications were filtered or masked, as this may substantially affect apparent CNVR coverage.

**Author response:** Thank you for the insightful comment. The reported value (~6.23%) represents the proportion of the autosomal genome covered by the union of non-redudant CNVR regions calculated by summing the total genomic length spanned by merged CNVRs and dividing it by the total autosomal genome size of the reference assembly. With regard to genomic context, this estimate is generally consistent with previous cattle CNV studies, which have reported CNVR coverage ranging from approximately 4-12% of the bovine genome depending on sequencing depth, detection methods and filtering strategies.

Regarding the repetitive and low mappability regions, CNV calling was performed using standard CNVnator and CNVcaller filtering procedures, which inherently reduce spurious calls in highly repetitive regions through read-depth normalization and quality filtering. However, no additional hard masking of repetitive regions or segmental duplication regions was applied prior to CNV detection. We acknowledge that the inclusion of such regions may influence CNVR coverage estimates.

**Reviewer Comment:** The reported mean CNVR length of about 27 kb, together with the statement that many CNVRs are under 5 kb, suggests a skewed size distribution. It would be more informative to provide the median CNVR size.

**Author response:** Thank you for this helpful suggestion. We agree that CNVR size distributions are typically skewed, and that the median provides a more informative measure of central tendency than mean in such cases. We have now included the median CNVR size in the manuscript which is 47 kb. This confirms a right-skewed distribution of CNVR lengths, characterized by a large number of smaller CNVRs and a small proportion of larger events.

**Reviewer Comment:** The comparison of CNVR counts across chromosomes should be normalized by chromosome length, as larger chromosomes are expected to harbour more CNVs. Without normalization, these comparisons are difficult to interpret.

Author response: Thank you for this comment. We agree that raw CNVR counts are influenced by chromosome length and may not be directly comparable across chromosomes. To address this, we have normalized CNVR counts by chromosome length and now report CNVR density (number of CNVRs per Mb) in the revised manuscript (Figure 1c).

**Reviewer Comment:** At lines 260–276, the manuscript interprets pairwise VST values across breeds. The use of V_ST_ is appropriate for CNV-based population differentiation, but the observed values indicate predominantly low to modest differentiation. Most pairwise comparisons are below 0.02, and even the highest value, around 0.084, is more consistent with moderate than “pronounced” differentiation. The language used in this section should therefore be toned down.

**Author response:** Thank you for this important observation. We agree that the magnitude of the reported V_ST_ values reflect predominantly low to moderate levels of population differentiation rather than pronounced divergence. The highlighted paragraph has been revised.

**Reviewer Comment:** In addition, statements that differentiation is “driven by region-specific CNVRs” are not directly demonstrated by the current analysis, because the manuscript does not present a locus-level analysis of the CNVRs contributing most strongly to VST. The authors should contextualize the observed VST range relative to other CNV studies, avoid overinterpreting modest values, and, if possible, identify specific high-VST CNVRs. A comparison with SNP-based population structure, if such data are available, would also strengthen interpretation.

**Author response:** Thank you for the comment. We agree that the statement implying that differentiation is ‘driven by region-specific CNVRs’ was not directly supported by a locus-level analysis and therefore been removed or revised in the manuscript. We also acknowledge that the current analysis does not include a CNVR-level decomposition of VST or a comparison with SNP-based population structure, which would provide additional resolution and is beyond the scope of the present study. To address this, we have revised the interpretation to ensure that the conclusions are limited to what our data directly supports.

**Reviewer Comment:** At lines 278–293, the manuscript reports a striking excess of breed-specific CNVRs in Hallikar relative to the other breeds. This is potentially interesting, but the degree of skew requires additional scrutiny. It is not clear whether this pattern reflects genuine biological divergence or whether it may be influenced by other factors. The authors should provide more support for this observation.

**Author response:** Thank you for the valuable comment. We agree with the comment, we have revised the manuscript to reflect a cautious interpretation and removed the speculative statements attributing this pattern to adaptation or selection, to avoid overstatement.

**Reviewer Comment:** The definition of “breed-specific” CNVRs should also be interpreted cautiously. The absence of detection in other breeds is not equivalent to a confirmed biological absence, especially in CNV analyses based on short-read read depth. Furthermore, statements linking Hallikar-specific CNVRs to local adaptation or selection are currently speculative and should be presented with greater caution unless stronger functional or population-genetic evidence is provided. The discussion should also explicitly note that CNVcaller filtering parameters may bias results toward shared CNVRs while reducing the detection of rare variants.

**Author response:** Thank you for the comment. We agree that the term ‘breed-specific CNVRs’ should be interpreted with caution in the context of short-read, read-depth based CNV detection, as absence of detection in other breeds does not necessarily imply true biological absence. We have revised the manuscript to clarify this limitation and to avoid overinterpretation. In addition, we have expanded the discussion to acknowledge the limitations posed by read depth-based approaches.

**Reviewer Comment:** Relating to lines 294-348, the author should specify the background gene set used in ShinyGO and clarify what is qualified as a gene “overlap” with a CNVR, because CNVRs can be large and overlap multiple genes. The analysis is also vulnerable to region-size bias, which should be acknowledged.

**Author response:** Thank you for the comment. The methodological details of functional enrichment analysis have been clarified in the revised manuscript. Specifically, we used a custom background gene set derived from the full gene annotation of the reference genome employed for CNVR mapping, rather than a default background provided by ShinyGO.

We also clarify that a gene was considered to overlap a CNVR if any portion of its genomic coordinates intersected the CNVR region. Given that the CNVRs may vary in size and can span multiple genes, we further acknowledge the potential region-size bias in enrichment results. This limitation has been added in the discussion.

**Reviewer Comment:** Moreover, the interpretation of the enrichment results is too strong. Broad categories such as immune regulation, metabolism, neuronal signalling, stress response, and endocrine pathways are common outputs in genome-wide enrichment analyses and do not by themselves constitute evidence for draught performance, endurance, thermotolerance, temperament, or adaptive energy utilization. Several statements in the current Results section go beyond what the data support by implying functional causality or phenotype-level inference from general enrichment results alone.

**Authors’ Response:** We agree with the reviewer. We have substantially revised the Results section to avoid implying direct functional causality or phenotype-level associations from broad GO and KEGG enrichment categories. We have also clarified in the revised manuscript, that these enrichments provide functional context for the CNVR-overlapping genes. rather than direct evidence of adaptive or production related phenotypes.

**Reviewer Comment:** The same caution applies to the QTL overlap analysis. Reporting overlap with QTL intervals can be useful as exploratory context, but the current manuscript does not establish whether the observed overlaps exceed chance expectation. The biological interpretation of these overlaps should be framed as hypothesis-generating rather than confirmatory. This is especially important for the Hallikar-specific CNVRs, where the number of events is relatively small, but the interpretation is still expansive.

**Authors’ Response:** Thanks for the comment. The manuscript is now revised, and the text avoids inferring direct biological significance from QTL intersections alone. The revised section now emphasizes that the analysis primarily identifies CNVRs located within genomic intervals previously reported to be associated with economically important cattle traits.

**Reviewer Comment:** Importantly, I strongly encourage the authors to shorten this section, focus on the most credible and biologically plausible candidate genes or pathways, clearly separate statistical enrichment from functional inference, and substantially tone down causal language.

**Authors’ Response:** Thanks for the suggestion. The text is now shortened and the needful done. The revised Results section has been made more concise, descriptive, and appropriately cautious in its interpretation of the findings.

**DISCUSSION**

**Reviewer Comment:** At lines 382–414, parts of the discussion read more like background material and may be more appropriate in the introduction. The discussion would be stronger if it focused more directly on what this dataset adds relative to prior reports.

**Authors Response:** We agree with the reviewer. The background-oriented portions and the generalized contextual statements in the Discussion section are removed in the revised manuscript. The Discussion has been revised to focus more specifically on the comparative significance of the present dataset and its contribution relative to previous CNV studies in *Bos indicus* cattle.

**Reviewer Comment:** At lines 402–404, if the study is framed as expanding previous datasets or knowledge, the discussion should explicitly state what is concordant with prior work and what is new here.

**Authors Response:** Agreed and Needful done.

**Reviewer Comment:** At lines 415–418, the restatement of the number of CNVs and CNVRs is clear, but the discussion should move beyond repeating results and instead interpret the implications more critically.

**Authors Response:** Agreed and Needful done.

**Kathiravan Periasamy**

**(Corresponding Author)**
