# Supplementary material for: Genome-wide characterization of copy number variants and their functional relevance in indigenous draught cattle of South Asia
Source: PLoS One. 2026 Jul 13;21(7):e0353468. doi: 10.1371/journal.pone.0353468 (PMC13362094; doi:10.1371/journal.pone.0353468)
Supplement: Attachment — Submitted filename: 1.Mavunga et al_Authors_Response_Reviewers.docx [file pone.0353468.s007.docx]

**Authors’ Response to Reviewers’ Comments**

**Academic Editor Comments**

1. Please deposit the custom scripts/workflows used for generating the CNVcaller duplicated-window files and reference database in a public repository and provide the repository URL. In addition, please include the repository link within the Data Availability Statement and associate URL of the BioProject accession.

**Authors’ Response:** We have deposited the custom scripts for generating the CNVcaller duplicated window files and reference database in the GitHub (<https://github.com/tafarakundai/cnvcaller-custom-reference-db>). The whole genome sequence data generated in the study are available in the form of paired end raw sequences (fastq.gz format) at NCBI under the bio-project accession number PRJNA1358578 (<https://www.ncbi.nlm.nih.gov/bioproject/?term=PRJNA1358578>). We also like to inform that the date of release of sequence data at NCBI has been set for 01 November 2026 or the date of publication of manuscript, whichever is earlier. The links to both the URLs are now indicated in the Data Availability Section during the submission of revision.

2. Please provide a sensitivity analysis (preferably as Supplementary Material) demonstrating the effect of different overlap thresholds on CNVR detection and the stability of the main conclusions.

**Authors’ Response:** A sensitivity analysis was performed using reciprocal overlap thresholds of 30%, 50%, 70%, and 90% for CNVR construction. The total number of detected CNVRs varied only modestly across thresholds, ranging from 5,992 at 30% overlap to 6,536 at 90% overlap. Relative to the 50% threshold used in the primary analysis, CNVR counts differed by −2.46%, +2.02%, and +6.40% at 30%, 70%, and 90%, respectively. Similar trends were observed for total genomic coverage and CNVR classification categories, indicating that the overall CNVR landscape was robust to the choice of overlap threshold.

The 50% reciprocal overlap threshold was retained for the main analysis because it provides a balance between over-merging distinct CNV events at lower thresholds and excessive fragmentation of CNVRs at higher thresholds. Furthermore, a 50% reciprocal overlap criterion has been widely adopted in previous CNV studies (Keel et al. 2016; Letaief et al. 2017; Butty et al. 2020; Buggiotti et al. 2022; Braga et al. 2023), facilitating comparison with published datasets. These results demonstrate that the principal conclusions of the study are not sensitive to the overlap threshold selected for CNVR construction.

| Threshold | CNVR_count | Total_bp | Pure_deletion | Pure_duplication | Mixed |
| --- | --- | --- | --- | --- | --- |
| 0.3 | 5,992 | 167,110,108 | 3,025 | 1,410 | 1,557 |
| 0.5 | 6,143 | 170,730,457 | 3,048 | 1,480 | 1,615 |
| 0.7 | 6,267 | 173,818,533 | 3,082 | 1,534 | 1,651 |
| 0.9 | 6,536 | 180,020,764 | 3,195 | 1,622 | 1,719 |

The above description and results of the sensitivity analysis is now included as Supporting Information File S4 in the revised version of the manuscript.

**References:**

1. Keel BN, Lindholm-Perry AK and Snelling WM (2016) Evolutionary and Functional Features of Copy Number Variation in the Cattle Genome. Front. Genet. 7:207. doi: 10.3389/fgene.2016.00207
2. Letaief, R., Rebours, E., Grohs, C. *et al.* Identification of copy number variation in French dairy and beef breeds using next-generation sequencing. *Genet Sel Evol* **49**, 77 (2017). <https://doi.org/10.1186/s12711-017-0352-z>
3. Butty, A.M., Chud, T.C.S., Miglior, F. *et al.* High confidence copy number variants identified in Holstein dairy cattle from whole genome sequence and genotype array data. *Sci Rep* **10**, 8044 (2020). <https://doi.org/10.1038/s41598-020-64680-3>
4. Buggiotti, L., Yudin, N. S., & Larkin, D. M. (2022). Copy Number Variants in Two Northernmost Cattle Breeds Are Related to Their Adaptive Phenotypes. Genes, 13(9), 1595. <https://doi.org/10.3390/genes13091595>
5. Braga, L. G., S. Chud, T. C., Watanabe, R. N., Savegnago, R. P., Sena, T. M., Machado, M. A., Panetto, C., & Munari, D. P. (2023). Identification of copy number variations in the genome of Dairy Gir cattle. *PLOS ONE*, *18*(4), e0284085. <https://doi.org/10.1371/journal.pone.0284085>

3. The substantially higher number of Hallikar-specific CNVRs warrants additional validation. Please provide evidence that this observation is not attributable to differences in sequencing depth, data quality, or CNV calling/filtering procedures.

**Authors’ Response:** We thank the Editor for this important comment regarding the higher number of Hallikar-specific CNVRs and the possibility of technical artefacts. To assess whether this observation could be attributed to differences in sequencing depth, data quality, or CNV calling procedures, we performed multiple validation checks across all samples and breeds.

- First, sequencing quality metrics (Table 1) showed no substantial deviation for Hallikar samples compared to other breeds. Mean sequencing depth and mapping quality were comparable across all datasets (Hallikar: ~18.5–21.9× coverage; mapping rate ~99.5–99.9%), indicating that differences in CNVR counts are not explained by sequencing depth or read quality.
- Second, CNVnator-derived raw CNV calls were compared across breeds. While variability in CNV counts was observed across all individuals—as expected for population-level CNV analysis - Hallikar samples did not show a consistent or systematic inflation relative to other breeds, and similar ranges of CNV counts were observed in other groups as well (please see the table below).
- Third, we examined CNVcaller intermediate outputs prior to merging. Hallikar also had similar number of initial window segments ~22k.

All samples were processed using identical parameters, normalization procedures, and filtering thresholds; therefore, downstream CNVR construction was performed uniformly across all breeds. Taken together, the consistency in sequencing metrics, the absence of systematic inflation in raw CNV counts across independent callers, and the uniform application of CNV filtering and merging criteria indicate that the higher number of Hallikar-specific CNVRs is not attributable to sequencing quality or methodological inconsistency but reflects variations in CNVs at individual animal level. For example, Table 3 shows that Hallikar had higher total and median CNVR lengths than the other breeds examined. This difference was primarily due to a greater number of duplication CNVRs identified in Hallikar. In contrast, the numbers of deletion and mixed CNVRs were comparable to those observed in the other breeds. These observations suggest that the higher number of Hallikar-specific CNVRs is unlikely to be an artefact of differences in CNV detection or filtering procedures.

**CNVnator output for Hallikar against other breeds:**

| Sample | Hallikar | Kangayam | Kdarm Red | White Cattle |
| --- | --- | --- | --- | --- |
| 1 | 4,892 | 4,365 | 4,127 | 5,223 |
| 2 | 6,141 | 4,242 | 4,241 | 5,285 |
| 3 | 4,631 | 4,168 | 5,626 | 4,127 |
| 4 | 9,253 | 4,247 | 4,179 | 4,594 |
| 5 | 5,819 | 4,199 | 3,967 | 4,119 |

4. Please provide complete details of the enrichment analysis, including the background gene set, annotation source/version, and significance thresholds used.

**Authors’ Response:** We thank the Editor for requesting clarification regarding the enrichment analysis background and annotation details.Functional enrichment analysis was performed using ShinyGO. Since ShinyGO does not currently provide a dedicated annotation database for *Bos indicus*, we selected the closest available and well-annotated reference, *Bos taurus* (ARS-UCD1.2; Ensembl/STRING-db ID: btaurus_gene_ensembl; Taxonomy ID: 9913), for pathway and Gene Ontology term mapping. However, to ensure species-appropriate statistical inference, we defined the background gene set using all genes annotated in the *Bos indicus* NIAB GTF file derived from the reference genome (GCF_029378745.1).

<https://ftp.ncbi.nlm.nih.gov/genomes/all/GCF/029/378/745/GCF_029378745.1_NIAB-ARS_B.indTharparkar_mat_pri_1.0/GCF_029378745.1_NIAB-ARS_B.indTharparkar_mat_pri_1.0_genomic.gtf.gz>

This ensured that enrichment analyses were performed relative to the complete gene universe of the indicine genome used in this study, rather than relying on the default *Bos taurus* background. The list of background gene set used to perform enrichment analysis is now provided as Supporting Information File S3 in the revised manuscript. For significance thresholds, we applied the following criteria and is already included in the Methodology Section of the manuscript:

ALL_CNVRS_GOBP: FDR < 0.01 and fold enrichment (FE) > 2.5

Core_CNVRS_GOBP: FDR < 0.01 and FE > 2.5

HAL_CNVRS_GOBP: FDR < 0.01 and FE > 1.5

**Journal Requirements:**

If the reviewer comments include a recommendation to cite specific previously published works, please review and evaluate these publications to determine whether they are relevant and should be cited. There is no requirement to cite these works unless the editor has indicated otherwise.

**Authors Response:** Not Applicable

Please review your reference list to ensure that it is complete and correct. If you have cited papers that have been retracted, please include the rationale for doing so in the manuscript text, or remove these references and replace them with relevant current references. Any changes to the reference list should be mentioned in the rebuttal letter that accompanies your revised manuscript. If you need to cite a retracted article, indicate the article’s retracted status in the References list and also include a citation and full reference for the retraction notice.

**Authors Response:** The reference list was checked and found correct.
