# Supplementary material for: Genome-wide characterization of copy number variants and their functional relevance in indigenous draught cattle of South Asia
Source: PLoS One. 2026 Jul 13;21(7):e0353468. doi: 10.1371/journal.pone.0353468 (PMC13362094; doi:10.1371/journal.pone.0353468)
Supplement: Peer review history

# Peer review: Decision Letter 0

**DOI:** 10.1371/journal.pone.0353468.r001

**Authors:** Tofazzal Md Rakib

16 Apr 2026

PONE-D-26-03707

Genome-wide characterization of copy number variants and their functional relevance in indigenous draught cattle of South Asia

PLOS One

Dear Dr. Periasamy,

Thank you for submitting your manuscript to PLOS ONE. After careful consideration, we feel that it has merit but does not fully meet PLOS ONE’s publication criteria as it currently stands. Therefore, we invite you to submit a revised version of the manuscript that addresses the points raised during the review process.

Please submit your revised manuscript by May 31 2026 11:59PM. If you will need more time than this to complete your revisions, please reply to this message or contact the journal office at plosone@plos.org. When you're ready to submit your revision, log on to https://www.editorialmanager.com/pone/ and select the 'Submissions Needing Revision' folder to locate your manuscript file.

Please include the following items when submitting your revised manuscript:

- A letter that responds to each point raised by the academic editor and reviewer(s). You should upload this letter as a separate file labeled 'Response to Reviewers'.
- A marked-up copy of your manuscript that highlights changes made to the original version. You should upload this as a separate file labeled 'Revised Manuscript with Track Changes'.
- An unmarked version of your revised paper without tracked changes. You should upload this as a separate file labeled 'Manuscript'.

If you would like to make changes to your financial disclosure, please include your updated statement in your cover letter. Guidelines for resubmitting your figure files are available below the reviewer comments at the end of this letter.

If applicable, we recommend that you deposit your laboratory protocols in protocols.io to enhance the reproducibility of your results. Protocols.io assigns your protocol its own identifier (DOI) so that it can be cited independently in the future. For instructions see: https://journals.plos.org/plosone/s/submission-guidelines#loc-laboratory-protocols. Additionally, PLOS ONE offers an option for publishing peer-reviewed Lab Protocol articles, which describe protocols hosted on protocols.io. Read more information on sharing protocols at https://plos.org/protocols?utm_medium=editorial-email&utm_source=authorletters&utm_campaign=protocols.

As the corresponding author, your ORCID iD is verified in the submission system and will appear in the published article. PLOS supports the use of ORCID, and we encourage all coauthors to register for an ORCID iD and use it as well. Please encourage your coauthors to verify their ORCID iD within the submission system before final acceptance, as unverified ORCID iDs will not appear in the published article. *Only*  the individual author can complete the verification step; PLOS staff *cannot*  verify ORCID iDs on behalf of authors.

We look forward to receiving your revised manuscript.

Kind regards,

Tofazzal Md Rakib, PhD

Academic Editor

PLOS One

Journal Requirements:

When submitting your revision, we need you to address these additional requirements.

1. Please ensure that your manuscript meets PLOS ONE's style requirements, including those for file naming. The PLOS ONE style templates can be found at

https://journals.plos.org/plosone/s/file?id=wjVg/PLOSOne_formatting_sample_main_body.pdf and

https://journals.plos.org/plosone/s/file?id=ba62/PLOSOne_formatting_sample_title_authors_affiliations.pdf.

2. Thank you for stating the following financial disclosure:

“International Atomic Energy Agency Technical cooperation projects KAM5009, SRL5046 and MYA5022.”

Please state what role the funders took in the study.  If the funders had no role, please state: "The funders had no role in study design, data collection and analysis, decision to publish, or preparation of the manuscript."

If this statement is not correct you must amend it as needed.

Please include this amended Role of Funder statement in your cover letter; we will change the online submission form on your behalf.

3. If the reviewer comments include a recommendation to cite specific previously published works, please review and evaluate these publications to determine whether they are relevant and should be cited. There is no requirement to cite these works unless the editor has indicated otherwise.

[Note: HTML markup is below. Please do not edit.]

Reviewers' comments:

Reviewer's Responses to Questions

**Comments to the Author**

1. Is the manuscript technically sound, and do the data support the conclusions?

The manuscript must describe a technically sound piece of scientific research with data that supports the conclusions. Experiments must have been conducted rigorously, with appropriate controls, replication, and sample sizes. The conclusions must be drawn appropriately based on the data presented.

Reviewer #1: Yes

Reviewer #2: Partly

**********

2. Has the statistical analysis been performed appropriately and rigorously?

Reviewer #1: Yes

Reviewer #2: No

**********

3. Have the authors made all data underlying the findings in their manuscript fully available?

The PLOS Data policy requires authors to make all data underlying the findings described in their manuscript fully available without restriction, with rare exception (please refer to the Data Availability Statement in the manuscript PDF file). The data should be provided as part of the manuscript or its supporting information, or deposited to a public repository. For example, in addition to summary statistics, the data points behind means, medians and variance measures should be available. If there are restrictions on publicly sharing data—e.g. participant privacy or use of data from a third party—those must be specified.

Reviewer #1: Yes

Reviewer #2: No

**********

4. Is the manuscript presented in an intelligible fashion and written in standard English?

PLOS ONE does not copyedit accepted manuscripts, so the language in submitted articles must be clear, correct, and unambiguous. Any typographical or grammatical errors should be corrected at revision, so please note any specific errors here.

Reviewer #1: Yes

Reviewer #2: Yes

**********

5. Review Comments to the Author

Please use the space provided to explain your answers to the questions above. You may also include additional comments for the author, including concerns about dual publication, research ethics, or publication ethics. (Please upload your review as an attachment if it exceeds 20,000 characters)

Reviewer #1: The manuscript's topic is of interest. The study provides valuable insights into the genome-wide characterization of copy number variation regions (CNVRs) in eight indigenous Asian zebu cattle, traditionally used for ploughing, wet-field agriculture, and carting. Several genes overlapping these CNVRs were associated with immune function, metabolism, and adaptation, and some regions corresponded to QTLs linked to carcass, fertility, reproduction, and growth traits. The findings provide important genomic resources for understanding structural variation and support cattle breeding and conservation programs. Overall, the manuscript is well-structured, and the findings are supported by vigorous data analysis.

Minor revision

• Introduction lines 100-128, the examples of breeds are lengthy. Consider shortening these examples to make the introduction more concise.

• Lines 153–155: “eight distinct breeds was performed with five samples per breed: Kangayam, Hallikar, Bargur and Deoni from India, White Cattle from Sri Lanka, Kdarm Red from Cambodia, Pyar Sein and Shwe Ni cattle from Myanmar” could be presented in a table format (e.g., breed name, country of origin, and number of samples). This would make the information easier to read.

Reviewer #2: This manuscript presents a genome-wide characterization of copy number variation regions across eight indigenous South Asian zebu cattle breeds using read-depth-based approaches, followed by analyses of population differentiation, functional annotation, and QTL overlap. The topic is relevant, and the dataset has value, particularly because structural variation remains undercharacterized in many indigenous cattle populations. The use of two CNV callers, genome-wide cataloging, and downstream functional analyses provides a potentially useful baseline resource.

Overall, I find the study technically sound, with an acceptable methodology but limited biological depth and consistent overinterpretation of results. The study is suitable for publication after major revision. My main concerns center on methodological justification, reporting clarity, and overinterpretation of the biological significance of the results. In particular, the CNV detection framework is broadly reasonable but requires additional explanation in several places; the population differentiation results are modest and should be interpreted more cautiously; the Hallikar-specific findings need stronger support; and the enrichment/QTL sections currently read as overly descriptive and more speculative than the data warrant.

Below are my specific major comments

Introduction:

• At lines 68-78, the discussion of CNVs, their size range, mechanisms of origin, and effects on gene expression should be more fully referenced.

• The section around lines 90-94 and 100-109 also needs supporting citations, especially where the manuscript summarizes the major computational approaches for CNV detection and their advantages.

• The breed descriptions at lines 105-120 could also be improved. A concise breed reference may be sufficient in the main text, while more detailed breed descriptions or representative photos could be moved to the Supplementary Information if the authors wish to provide additional context.

• 132-134, The manuscript would benefit from a more focused introduction that clearly summarizes what is currently known about CNVs in Asian zebu cattle. This should include key findings from previous studies, commonly used methodologies, and any existing limitations in the literature. In addition, the authors should explicitly define the specific gap this study addresses and the new insights it aims to provide. Clarifying this will help the reader better understand the motivation and the precise contribution of the work.

Materials and methods:

• At lines 155–159, the manuscript should provide more explicit information about the source of the DNA samples, how genomic DNA was extracted or processed, and the sequencing design. It is also important to state whether the reads were single-end or paired-end, their read length, and the target or achieved sequencing depth per sample. These details are necessary for reproducibility and for evaluating the suitability of the data for read-depth–based CNV calling.

• The use of CNVnator and CNVcaller is a reasonable approach for CNV discovery from short-read data. However, several aspects of the methodology here still need clarification and justification. For instance, at lines 179–180, the authors should justify the use of a 100 bp bin size in CNVnator. This choice increases nominal resolution but can also increase susceptibility to local coverage noise. It would also be helpful to know whether alternative bin sizes were considered and why 100 bp was ultimately selected.

• At lines 195–197, the generation of custom duplicated window files and a custom reference setup for CNVcaller introduces reproducibility concerns. The authors should describe more clearly how this resource was generated and validated, and ideally make the pipeline or script publicly available.

• At lines 201–205, the CNVcaller filters -f 0.1 and -h 3 may bias the analysis toward common CNVs while excluding low-frequency or truly breed-specific events. This is particularly relevant because the manuscript later interprets the biological significance of shared versus breed-specific CNVRs. The authors should discuss how these thresholds may influence the observed CNVR spectrum and whether they reduce sensitivity to rare variants.

• At line 204, the use of a 50% reciprocal overlap criterion for merging CNVs into CNVRs should be explicitly justified. This is a common heuristic, but it remains an arbitrary threshold that can affect the number and size of CNVRs. If possible, a brief sensitivity check or rationale would strengthen the methodological rigor.

Results:

• At line 236, the reported sequencing metrics, including mean coverage around 21.2× and mapping rate around 99%, indicate good overall data quality and support the use of read-depth–based CNV detection in this dataset. However, the manuscript’s wording that this enables “accurate CNV detection” is somewhat too strong given the known limitations of read-depth methods, especially in repetitive or low-mappability regions. A more cautious phrasing would be appropriate.

• At lines 244–245, the statement that CNVs were retained based on “p-value < 0.01 based on t-test statistics” requires more detail. CNVnator reports multiple p-value-related metrics, and it is currently unclear which statistic the authors used. The manuscript should clarify which CNVnator p-value output was applied, how that p-value is defined within CNVnator, and whether any multiple-testing considerations were taken into account. The wording should also make clear that this is part of CNVnator’s internal model rather than a separate test performed independently by the authors.

• At lines 251–259, the manuscript reports that CNVRs cover approximately 6.23% of the autosomal genome. This should be contextualized against previous cattle CNV studies. The authors should also clarify whether repetitive regions or segmental duplications were filtered or masked, as this may substantially affect apparent CNVR coverage.

• The reported mean CNVR length of about 27 kb, together with the statement that many CNVRs are under 5 kb, suggests a skewed size distribution. It would be more informative to provide the median CNVR size.

• The comparison of CNVR counts across chromosomes should be normalized by chromosome length, as larger chromosomes are expected to harbor more CNVs. Without normalization, these comparisons are difficult to interpret.

• At lines 260–276, the manuscript interprets pairwise VST values across breeds. The use of VST is appropriate for CNV-based population differentiation, but the observed values indicate predominantly low to modest differentiation. Most pairwise comparisons are below 0.02, and even the highest value, around 0.084, is more consistent with moderate than “pronounced” differentiation. The language used in this section should therefore be toned down.

• In addition, statements that differentiation is “driven by region-specific CNVRs” are not directly demonstrated by the current analysis, because the manuscript does not present a locus-level analysis of the CNVRs contributing most strongly to VST. The authors should contextualize the observed VST range relative to other CNV studies, avoid overinterpreting modest values, and, if possible, identify specific high-VST CNVRs. A comparison with SNP-based population structure, if such data are available, would also strengthen interpretation.

• At lines 278–293, the manuscript reports a striking excess of breed-specific CNVRs in Hallikar relative to the other breeds. This is potentially interesting, but the degree of skew requires additional scrutiny. It is not clear whether this pattern reflects genuine biological divergence or whether it may be influenced by other factors. The authors should provide more support for this observation.

• The definition of “breed-specific” CNVRs should also be interpreted cautiously. The absence of detection in other breeds is not equivalent to a confirmed biological absence, especially in CNV analyses based on short-read read depth. Furthermore, statements linking Hallikar-specific CNVRs to local adaptation or selection are currently speculative and should be presented with greater caution unless stronger functional or population-genetic evidence is provided. The discussion should also explicitly note that CNVcaller filtering parameters may bias results toward shared CNVRs while reducing the detection of rare variants.

• Relating to lines 294-348, the author should specify the background gene set used in ShinyGO and clarify what is qualified as a gene “overlap” with a CNVR, because CNVRs can be large and overlap multiple genes. The analysis is also vulnerable to region-size bias, which should be acknowledged.

• Moreover, the interpretation of the enrichment results is too strong. Broad categories such as immune regulation, metabolism, neuronal signaling, stress response, and endocrine pathways are common outputs in genome-wide enrichment analyses and do not by themselves constitute evidence for draught performance, endurance, thermotolerance, temperament, or adaptive energy utilization. Several statements in the current Results section go beyond what the data support by implying functional causality or phenotype-level inference from general enrichment results alone.

• The same caution applies to the QTL overlap analysis. Reporting overlap with QTL intervals can be useful as exploratory context, but the current manuscript does not establish whether the observed overlaps exceed chance expectation. The biological interpretation of these overlaps should be framed as hypothesis-generating rather than confirmatory. This is especially important for the Hallikar-specific CNVRs, where the number of events is relatively small but the interpretation is still expansive.

• Importantly, I strongly encourage the authors to shorten this section, focus on the most credible and biologically plausible candidate genes or pathways, clearly separate statistical enrichment from functional inference, and substantially tone down causal language.

Discussion

• At lines 382–414, parts of the discussion read more like background material and may be more appropriate in the introduction. The discussion would be stronger if it focused more directly on what this dataset adds relative to prior reports.

• At lines 402–404, if the study is framed as expanding previous datasets or knowledge, the discussion should explicitly state what is concordant with prior work and what is new here.

• At lines 415–418, the restatement of the number of CNVs and CNVRs is clear, but the discussion should move beyond repeating results and instead interpret the implications more critically.

**********

6. PLOS authors have the option to publish the peer review history of their article (what does this mean?). If published, this will include your full peer review and any attached files.

If you choose “no”, your identity will remain anonymous but your review may still be made public.

**Do you want your identity to be public for this peer review?**  For information about this choice, including consent withdrawal, please see our Privacy Policy.

Reviewer #1: No

Reviewer #2: No

**********

[NOTE: If reviewer comments were submitted as an attachment file, they will be attached to this email and accessible via the submission site. Please log into your account, locate the manuscript record, and check for the action link "View Attachments". If this link does not appear, there are no attachment files.]

To ensure your figures meet our technical requirements, please review our figure guidelines: https://journals.plos.org/plosone/s/figures

You may also use PLOS’s free figure tool, NAAS, to help you prepare publication quality figures: https://journals.plos.org/plosone/s/figures#loc-tools-for-figure-preparation.

NAAS will assess whether your figures meet our technical requirements by comparing each figure against our figure specifications.

# Author comment: Author response to Decision Letter 1

**DOI:** 10.1371/journal.pone.0353468.r002

25 May 2026

RESPONSE TO REVIEWERS’ COMMENTS

EDITOR’S COMMENTS

Thank you for submitting your manuscript to PLOS ONE. After careful consideration, we feel that it has merit but does not fully meet PLOS ONE’s publication criteria as it currently stands. Therefore, we invite you to submit a revised version of the manuscript that addresses the points raised during the review process.

Authors’ Response: Thanks for the comment. The manuscript is now revised as per the comments of both the reviewers

REVIEWER 1 COMMENTS

The manuscript's topic is of interest. The study provides valuable insights into the genome-wide characterization of copy number variation regions (CNVRs) in eight indigenous Asian zebu cattle, traditionally used for ploughing, wet-field agriculture, and carting. Several genes overlapping these CNVRs were associated with immune function, metabolism, and adaptation, and some regions corresponded to QTLs linked to carcass, fertility, reproduction, and growth traits. The findings provide important genomic resources for understanding structural variation and support cattle breeding and conservation programs. Overall, the manuscript is well-structured, and the findings are supported by vigorous data analysis.

Authors’ Response: We thank the reviewer for their positive evaluation of the manuscript and for their overall comment.

Reviewer 1 Comment: Introduction lines 100-128, the examples of breeds are lengthy. Consider shortening these examples to make the introduction more concise.

Authors’ Response: Thank you for the comment, we have shortened examples of breeds to allow conciseness of the introduction.

Reviewer 1 Comment: Lines 153–155: “eight distinct breeds was performed with five samples per breed: Kangayam, Hallikar, Bargur and Deoni from India, White Cattle from Sri Lanka, Kdarm Red from Cambodia, Pyar Sein and Shwe Ni cattle from Myanmar” could be presented in a table format (e.g., breed name, country of origin, and number of samples). This would make the information easier to read.

Authors’ Response: We thank the reviewer for their comment, we have included the table in the manuscript at suggested.

REVIEWER 2 COMMENTS

This manuscript presents a genome-wide characterization of copy number variation regions across eight indigenous South Asian zebu cattle breeds using read-depth-based approaches, followed by analyses of population differentiation, functional annotation, and QTL overlap. The topic is relevant, and the dataset has value, particularly because structural variation remains under characterized in many indigenous cattle populations. The use of two CNV callers, genome-wide cataloguing, and downstream functional analyses provides a potentially useful baseline resource.

Overall, I find the study technically sound, with an acceptable methodology but limited biological depth and consistent overinterpretation of results. The study is suitable for publication after major revision. My main concerns center on methodological justification, reporting clarity, and overinterpretation of the biological significance of the results. In particular, the CNV detection framework is broadly reasonable but requires additional explanation in several places; the population differentiation results are modest and should be interpreted more cautiously; the Hallikar-specific findings need stronger support; and the enrichment/QTL sections currently read as overly descriptive and more speculative than the data warrant.

Author Response: We thank the reviewer for their positive evaluation of the manuscript and for their overall comment. The main concerns/suggestions of the reviewer are addressed in the revised manuscript.

INTRODUCTION

Reviewer Comment: At lines 68-78, the discussion of CNVs, their size range, mechanisms of origin, and effects on gene expression should be more fully referenced.

Author Response: Thank you for the comment, we have adjusted the section and included the below references to support the statements.

Hastings, P., Lupski, J., Rosenberg, S. et al. Mechanisms of change in gene copy number. Nat Rev Genet 10, 551–564 (2009). https://doi.org/10.1038/nrg2593

Louise Harewood, Peter Fraser, The impact of chromosomal rearrangements on regulation of gene expression, Human Molecular Genetics, Volume 23, Issue R1, 15 September 2014, Pages R76–R82, https://doi.org/10.1093/hmg/ddu278

Eric R. Gamazon, Barbara E. Stranger, The impact of human copy number variation on gene expression, Briefings in Functional Genomics, Volume 14, Issue 5, September 2015, Pages 352–357, https://doi.org/10.1093/bfgp/elv017

Clop, A., Vidal, O. and Amills, M. (2012), Copy number variation in the genomes of domestic animals. Anim Genet, 43: 503-517. https://doi.org/10.1111/j.1365 2052.2012.02317.x

Fadista, J., Thomsen, B., Holm, LE. et al. Copy number variation in the bovine genome. BMC Genomics 11, 284 (2010). https://doi.org/10.1186/1471-2164-11-284

Reviewer Comment: The section around lines 90-94 and 100-109 also needs supporting citations, especially where the manuscript summarizes the major computational approaches for CNV detection and their advantages.

Author Response: Thank you for the comment we have added the below references to support section 90-94.

Zhao M, Wang Q, Wang Q, Jia P, Zhao Z. Computational tools for copy number variation (CNV) detection using next-generation sequencing data: features and perspectives. BMC Bioinformatics. 2013 Sep 13;14(S11):S1. doi:10.1186/1471-2105-14-S11-S1

Zhang ZD, Du J, Lam H, Abyzov A, Urban AE, Snyder M, et al. Identification of genomic indels and structural variations using split reads. BMC Genomics. 2011 Dec 25;12(1):375. doi:10.1186/1471-2164-12-375

Pirooznia M, Goes FS, Zandi PP. Whole-genome CNV analysis: advances in computational approaches. Front Genet. 2015 Apr 13;06.doi:10.3389/fgene.2015.00138

Whitford W, Lehnert K, Snell RG, Jacobsen JC. Evaluation of the performance of copy number variant prediction tools for the detection of deletions from whole genome sequencing data. J Biomed Inform. 2019 Jun;94:103174. doi:10.1016/j.jbi.2019.103174

Yoon S, Xuan Z, Makarov V, Ye K, Sebat J. Sensitive and accurate detection of copy number variants using read depth of coverage. Genome Res. 2009 Sep;19(9):1586–92. doi:10.1101/gr.092981.109

Author Response: Thank you for the comment we have added the below references to support section 100-109. These references describe the functional uses of the breeds, their origins and characteristics.

Mavunga, T.K., Sölkner, J., Mészáros, G. et al. Genomic diversity and selection signatures in Asian Zebu Cattle: insights into adaptation and genetic erosion. Sci Rep 15, 33346 (2025). https://doi.org/10.1038/s41598-025-14744-z

Singh PK. Phenotypic characterization and performance evaluation of Hallikar cattle in its native tract. Indian Journal of Animal Sciences. 2008;78(2):211–4.

Kuralkar SV. Phenotypic characteristics, production and reproduction performance of Deoni cattle in its native tract. . Indian Journal of Animal Sciences,. 2014;84(1):75–7.

Hattarakihal M, Patil VM, Waghmare PG, Suranagi M, Kulkarni S, Desai AR, et al. Morphometric traits of different Deoni cattle strains. International Journal of Veterinary Sciences and Animal Husbandry. 2023 Jan 1;8(4S):151–4. doi:10.22271/veterinary.2023.v8.i4Sc.663

Lokugalappatti LGS, Wickramasinghe S, Alexander PABD, Abbas K, Hussain T, Ramasamy S, et al. Indigenous cattle of Sri Lanka: Genetic and phylogeographic relationship with Zebu of Indus Valley and South Indian origin. PLoS One. 2023 Aug 1;18(8 August). doi:10.1371/journal.pone.0282761 PubMed PMID: 37585485.

Lwin M, Mon SLY, Yamanaka H, Nagano Y, Mannen H, Faruque MO, et al. Genetic diversities and population structures of four popular Myanmar local cattle breeds. Animal Science Journal. 2018 Dec 14;89(12):1648–55. doi:10.1111/asj.13112

Namikawa T. MH, TK, NK, TY,. Coat-color variations of cattle observed in the fields of Cambodia, and withers-height and other traits in the native cattle subjected to further experimental analyses. Rep Soc Res Native Livestock. 2006; 23:31–44.

Reviewer Comment: The breed descriptions at lines 105-120 could also be improved. A concise breed reference may be sufficient in the main text, while more detailed breed descriptions or representative photos could be moved to the Supplementary Information if the authors wish to provide additional context.

Author Response: We thank the reviewer for this helpful suggestion. The breed descriptions in lines 105–120 have been revised to improve conciseness and readability. In the main text, we now provide only brief information on breed origin and key functional traits relevant to the study. More detailed descriptions have been removed to avoid redundancy and maintain focus.

Reviewer Comment: 132-134, The manuscript would benefit from a more focused introduction that clearly summarizes what is currently known about CNVs in Asian zebu cattle. This should include key findings from previous studies, commonly used methodologies, and any existing limitations in the literature. In addition, the authors should explicitly define the specific gap this study addresses and the new insights it aims to provide. Clarifying this will help the reader better understand the motivation and the precise contribution of the work.

Author Response: We thank the reviewer for this insightful suggestion. The Introduction has been revised to provide a clearer overview of copy number variations (CNVs) in Asian zebu cattle, with particular emphasis on South Asian and Southeast Asian populations. We have incorporated relevant literature describing CNV patterns in indicine cattle and outlined commonly used methodologies for CNV detection, including array-based and next-generation sequencing approaches.

In addition, we have highlighted key limitations in the existing literature, such as the underrepresentation of indigenous zebu breeds from South and Southeast Asia and challenges related to CNV detection and comparability across studies. Finally, we have clarified the specific knowledge gap addressed by this study and explicitly stated its contribution to improving the understanding of CNV diversity in South and Southeast Asian zebu cattle.

MATERIALS AND METHODS

Reviewer Comment: At lines 155–159, the manuscript should provide more explicit information about the source of the DNA samples, how genomic DNA was extracted or processed, and the sequencing design. It is also important to state whether the reads were single-end or paired-end, their read length, and the target or achieved sequencing depth per sample. These details are necessary for reproducibility and for evaluating the suitability of the data for read-depth–based CNV calling.

Author Response: Thanks for the comment and the opportunity to clarify our sample collection and DNA extraction strategy. All the DNA samples used in the present study were derived from the existing Genetic Repository at Animal Production and Health Laboratory, Joint FAO/IAEA Centre of Nuclear Techniques in Food and Agriculture, International Atomic Energy Agency, Vienna, Austria (Already indicated under the Ethics statement in the Methods section). The DNA available at the FAO/IAEA repository were extracted from blood samples collected by jugular venipuncture into EDTA vacutainer tubes and isolated using MasterPure DNA Purification Kit (Biozym, Illumina Inc, USA). It was ensured that the samples included in the study were collected from unrelated cattle, based on the information available in the Genetic Repository module of FAO/IAEA Genetic Laboratory Information and Data Management System (GLIDMaS). We have revised the manuscript to include the above information for better clarity. We have also added details of sequencing design, specifying that paired-end sequencing was used, along with read length. In addition, the average sequencing depth per sample, has been included in the revised version of the manuscript.

Reviewer Comment: The use of CNVnator and CNVcaller is a reasonable approach for CNV discovery from short-read data. However, several aspects of the methodology here still need clarification and justification. For instance, at lines 179–180, the authors should justify the use of a 100 bp bin size in CNVnator. This choice increases nominal resolution but can also increase susceptibility to local coverage noise. It would also be helpful to know whether alternative bin sizes were considered and why 100 bp was ultimately selected.

Author Response: Thank you for this valuable comment. We agree that precision in breakpoint location is a function of bin size in CNVnator. As per the developers of CNVnator (Abyzov et al. 2011), the choice of bin size is a function of coverage, read length, and data quality. Given the data quality and read length remaining same, the optimal bin size (and thus breakpoint resolution accuracy) scales inversely with the coverage. Hence, Abyzov et al (2011) recommended a bin size of ∼100-bp for sequence data with 20–30× coverage, ∼500-bp bins for 4–6× coverage, and ∼30-bp bins for ∼100× coverage. Hence, in the present study we chose a bin size of 100bp since the mean coverage of our sequence data was 21.63 ± 0.36.

Reviewer Comment: At lines 195–197, the generation of custom duplicated window files and a custom reference setup for CNVcaller introduces reproducibility concerns. The authors should describe more clearly how this resource was generated and validated and ideally make the pipeline or script publicly available.

Author response: Thank you for this comment. We like to clarify that the duplicated window files and reference database were generated strictly following the official CNVcaller pipeline provided by the developers (https://github.com/JiangYuLab/CNVcaller). Their inbuilt database is made from the taurine reference genome, and they provide steps to guide one to make their own custom database for subspecies like indicine. We have revised the Methods section to explicitly describe the key steps involved using the provided scripts. To further enhance reproducibility, we have provided exact commands used in this study under the Methods section in the revised manuscript enabling full replication of the workflow.

Reviewer Comment: At lines 201–205, the CNVcaller filters -f 0.1 and -h 3 may bias the analysis toward common CNVs while excluding low-frequency or truly breed-specific events. This is particularly relevant because the manuscript later interprets the biological significance of shared versus breed-specific CNVRs. The authors should discuss how these thresholds may influence the observed CNVR spectrum and whether they reduce sensitivity to rare variants.

Author response: We thank the reviewer for this comment. We agree that the choice of filtering thresholds in CNVcaller can influence the resulting CNVRs detected, and the balance between sensitivity and specificity. In our study we applied filtering thresholds as recommended in the CNVcaller framework (https://github.com/JiangYuLab/CNVcaller) to reduce false-positive CNV calls arising from stochastic read-depth variation and low-support signals, particularly given the ~20x sequencing depth used in this study. These parameters are commonly used to retain CNVs supported by a minimum proportion of individuals while ensuring robustness of detected variants (Deng TX, Ma XY, Duan A, Lu XR, Abdel-Shafy H. Genome-wide copy number variant analysis reveals candidate genes associated with milk production traits in water buffalo (Bubalus bubalis). J Dairy Sci. 2024 Sep;107(9):7022-7037. doi: 10.3168/jds.2023-24614) (Liu Y, Mu Y, Wang W, Ahmed Z, Wei X, Lei C and Ma Z (2023) Analysis of genomic copy number variations through whole-genome scan in Chinese Qaidam cattle. Front. Vet. Sci. 10:1148070. doi: 10.3389/fvets.2023.1148070). We acknowledge that these filtering criteria may reduce sensitivity to rare or low-frequency CNVs, including potentially breed-specific variants present at low prevalence. As a result, the CNVRs reported here represent a conservative set of high-confidence variants, and the interpretation of shred versus breed-specific CNVRs is focused on variants that pass these stringent filters. This limitation and its implications for the bias provided by the filters have been discus

Attachment Submitted filename: 1.Mavunga_Response_To_Reviewers.docx

# Peer review: Decision Letter 1

**DOI:** 10.1371/journal.pone.0353468.r003

**Authors:** Tofazzal Md Rakib, Tofazzal Md Rakib

17 Jun 2026

PONE-D-26-03707R1

Genome-wide characterization of copy number variants and their functional relevance in indigenous draught cattle of South Asia

PLOS One

Dear Dr. Periasamy,

Thank you for submitting your manuscript to PLOS ONE. After careful consideration, we feel that it has merit but does not fully meet PLOS ONE’s publication criteria as it currently stands. Therefore, we invite you to submit a revised version of the manuscript that addresses the points raised during the review process.

==============================

**Academic Editor Comments:**

1. Please deposit the custom scripts/workflows used for generating the CNVcaller duplicated-window files and reference database in a public repository and provide the repository URL. In addition, please include the repository link within the Data Availability Statement and associate URL of the BioProject accession.

2. Please provide a sensitivity analysis (preferably as Supplementary Material) demonstrating the effect of different overlap thresholds on CNVR detection and the stability of the main conclusions.

3. The substantially higher number of Hallikar-specific CNVRs warrants additional validation. Please provide evidence that this observation is not attributable to differences in sequencing depth, data quality, or CNV calling/filtering procedures.

4. Please provide complete details of the enrichment analysis, including the background gene set, annotation source/version, and significance thresholds used.

==============================

Please submit your revised manuscript by Aug 01 2026 11:59PM. If you will need more time than this to complete your revisions, please reply to this message or contact the journal office at plosone@plos.org. When you're ready to submit your revision, log on to https://www.editorialmanager.com/pone/ and select the 'Submissions Needing Revision' folder to locate your manuscript file.

Please include the following items when submitting your revised manuscript:

- A letter that responds to each point raised by the academic editor and reviewer(s). You should upload this letter as a separate file labeled 'Response to Reviewers'.
- A marked-up copy of your manuscript that highlights changes made to the original version. You should upload this as a separate file labeled 'Revised Manuscript with Track Changes'.
- An unmarked version of your revised paper without tracked changes. You should upload this as a separate file labeled 'Manuscript'.

If you would like to make changes to your financial disclosure, please include your updated statement in your cover letter. Guidelines for resubmitting your figure files are available below the reviewer comments at the end of this letter.

If applicable, we recommend that you deposit your laboratory protocols in protocols.io to enhance the reproducibility of your results. Protocols.io assigns your protocol its own identifier (DOI) so that it can be cited independently in the future. For instructions see: https://journals.plos.org/plosone/s/submission-guidelines#loc-laboratory-protocols. Additionally, PLOS ONE offers an option for publishing peer-reviewed Lab Protocol articles, which describe protocols hosted on protocols.io. Read more information on sharing protocols at https://plos.org/protocols?utm_medium=editorial-email&utm_source=authorletters&utm_campaign=protocols.

As the corresponding author, your ORCID iD is verified in the submission system and will appear in the published article. PLOS supports the use of ORCID, and we encourage all coauthors to register for an ORCID iD and use it as well. Please encourage your coauthors to verify their ORCID iD within the submission system before final acceptance, as unverified ORCID iDs will not appear in the published article. *Only*  the individual author can complete the verification step; PLOS staff *cannot*  verify ORCID iDs on behalf of authors.

We look forward to receiving your revised manuscript.

Kind regards,

Tofazzal Md Rakib, PhD

Academic Editor

PLOS One

**Journal Requirements:**

1. If the reviewer comments include a recommendation to cite specific previously published works, please review and evaluate these publications to determine whether they are relevant and should be cited. There is no requirement to cite these works unless the editor has indicated otherwise.

2. Please review your reference list to ensure that it is complete and correct. If you have cited papers that have been retracted, please include the rationale for doing so in the manuscript text, or remove these references and replace them with relevant current references. Any changes to the reference list should be mentioned in the rebuttal letter that accompanies your revised manuscript. If you need to cite a retracted article, indicate the article’s retracted status in the References list and also include a citation and full reference for the retraction notice.

[Note: HTML markup is below. Please do not edit.]

Reviewers' comments:

Reviewer's Responses to Questions

**Comments to the Author**

1. If the authors have adequately addressed your comments raised in a previous round of review and you feel that this manuscript is now acceptable for publication, you may indicate that here to bypass the “Comments to the Author” section, enter your conflict of interest statement in the “Confidential to Editor” section, and submit your "Accept" recommendation.

Reviewer #1: All comments have been addressed

**********

2. Is the manuscript technically sound, and do the data support the conclusions?

The manuscript must describe a technically sound piece of scientific research with data that supports the conclusions. Experiments must have been conducted rigorously, with appropriate controls, replication, and sample sizes. The conclusions must be drawn appropriately based on the data presented.

Reviewer #1: Yes

**********

3. Has the statistical analysis been performed appropriately and rigorously?

Reviewer #1: Yes

**********

4. Have the authors made all data underlying the findings in their manuscript fully available?

The PLOS Data policy requires authors to make all data underlying the findings described in their manuscript fully available without restriction, with rare exception (please refer to the Data Availability Statement in the manuscript PDF file). The data should be provided as part of the manuscript or its supporting information, or deposited to a public repository. For example, in addition to summary statistics, the data points behind means, medians and variance measures should be available. If there are restrictions on publicly sharing data—e.g. participant privacy or use of data from a third party—those must be specified.

Reviewer #1: Yes

**********

5. Is the manuscript presented in an intelligible fashion and written in standard English?

PLOS ONE does not copyedit accepted manuscripts, so the language in submitted articles must be clear, correct, and unambiguous. Any typographical or grammatical errors should be corrected at revision, so please note any specific errors here.

Reviewer #1: Yes

**********

6. Review Comments to the Author

Please use the space provided to explain your answers to the questions above. You may also include additional comments for the author, including concerns about dual publication, research ethics, or publication ethics. (Please upload your review as an attachment if it exceeds 20,000 characters)

Reviewer #1: (No Response)

**********

7. PLOS authors have the option to publish the peer review history of their article (what does this mean?). If published, this will include your full peer review and any attached files.

If you choose “no”, your identity will remain anonymous but your review may still be made public.

**Do you want your identity to be public for this peer review?**  For information about this choice, including consent withdrawal, please see our Privacy Policy.

Reviewer #1: No

**********

[NOTE: If reviewer comments were submitted as an attachment file, they will be attached to this email and accessible via the submission site. Please log into your account, locate the manuscript record, and check for the action link "View Attachments". If this link does not appear, there are no attachment files.]

To ensure your figures meet our technical requirements, please review our figure guidelines: https://journals.plos.org/plosone/s/figures

You may also use PLOS’s free figure tool, NAAS, to help you prepare publication quality figures: https://journals.plos.org/plosone/s/figures#loc-tools-for-figure-preparation.

NAAS will assess whether your figures meet our technical requirements by comparing each figure against our figure specifications.

# Author comment: Author response to Decision Letter 2

**DOI:** 10.1371/journal.pone.0353468.r004

19 Jun 2026

Authors’ Response to Reviewers’ Comments

Academic Editor Comments

1. Please deposit the custom scripts/workflows used for generating the CNVcaller duplicated-window files and reference database in a public repository and provide the repository URL. In addition, please include the repository link within the Data Availability Statement and associate URL of the BioProject accession.

Authors’ Response: We have deposited the custom scripts for generating the CNVcaller duplicated window files and reference database in the GitHub (https://github.com/tafarakundai/cnvcaller-custom-reference-db). The whole genome sequence data generated in the study are available in the form of paired end raw sequences (fastq.gz format) at NCBI under the bio-project accession number PRJNA1358578 (https://www.ncbi.nlm.nih.gov/bioproject/?term=PRJNA1358578). We also like to inform that the date of release of sequence data at NCBI has been set for 01 November 2026 or the date of publication of manuscript, whichever is earlier. The links to both the URLs are now indicated in the Data Availability Section during the submission of revision.

2. Please provide a sensitivity analysis (preferably as Supplementary Material) demonstrating the effect of different overlap thresholds on CNVR detection and the stability of the main conclusions.

Authors’ Response: A sensitivity analysis was performed using reciprocal overlap thresholds of 30%, 50%, 70%, and 90% for CNVR construction. The total number of detected CNVRs varied only modestly across thresholds, ranging from 5,992 at 30% overlap to 6,536 at 90% overlap. Relative to the 50% threshold used in the primary analysis, CNVR counts differed by −2.46%, +2.02%, and +6.40% at 30%, 70%, and 90%, respectively. Similar trends were observed for total genomic coverage and CNVR classification categories, indicating that the overall CNVR landscape was robust to the choice of overlap threshold.

The 50% reciprocal overlap threshold was retained for the main analysis because it provides a balance between over-merging distinct CNV events at lower thresholds and excessive fragmentation of CNVRs at higher thresholds. Furthermore, a 50% reciprocal overlap criterion has been widely adopted in previous CNV studies (Keel et al. 2016; Letaief et al. 2017; Butty et al. 2020; Buggiotti et al. 2022; Braga et al. 2023), facilitating comparison with published datasets. These results demonstrate that the principal conclusions of the study are not sensitive to the overlap threshold selected for CNVR construction.

Threshold CNVR_count Total_bp Pure_deletion Pure_duplication Mixed

0.3 5,992 167,110,108 3,025 1,410 1,557

0.5 6,143 170,730,457 3,048 1,480 1,615

0.7 6,267 173,818,533 3,082 1,534 1,651

0.9 6,536 180,020,764 3,195 1,622 1,719

The above description and results of the sensitivity analysis is now included as Supporting Information File S4 in the revised version of the manuscript.

References:

1. Keel BN, Lindholm-Perry AK and Snelling WM (2016) Evolutionary and Functional Features of Copy Number Variation in the Cattle Genome. Front. Genet. 7:207. doi: 10.3389/fgene.2016.00207

2. Letaief, R., Rebours, E., Grohs, C. et al. Identification of copy number variation in French dairy and beef breeds using next-generation sequencing. Genet Sel Evol 49, 77 (2017). https://doi.org/10.1186/s12711-017-0352-z

3. Butty, A.M., Chud, T.C.S., Miglior, F. et al. High confidence copy number variants identified in Holstein dairy cattle from whole genome sequence and genotype array data. Sci Rep 10, 8044 (2020). https://doi.org/10.1038/s41598-020-64680-3

4. Buggiotti, L., Yudin, N. S., & Larkin, D. M. (2022). Copy Number Variants in Two Northernmost Cattle Breeds Are Related to Their Adaptive Phenotypes. Genes, 13(9), 1595. https://doi.org/10.3390/genes13091595

5. Braga, L. G., S. Chud, T. C., Watanabe, R. N., Savegnago, R. P., Sena, T. M., Machado, M. A., Panetto, C., & Munari, D. P. (2023). Identification of copy number variations in the genome of Dairy Gir cattle. PLOS ONE, 18(4), e0284085. https://doi.org/10.1371/journal.pone.0284085

3. The substantially higher number of Hallikar-specific CNVRs warrants additional validation. Please provide evidence that this observation is not attributable to differences in sequencing depth, data quality, or CNV calling/filtering procedures.

Authors’ Response: We thank the Editor for this important comment regarding the higher number of Hallikar-specific CNVRs and the possibility of technical artefacts. To assess whether this observation could be attributed to differences in sequencing depth, data quality, or CNV calling procedures, we performed multiple validation checks across all samples and breeds.

• First, sequencing quality metrics (Table 1) showed no substantial deviation for Hallikar samples compared to other breeds. Mean sequencing depth and mapping quality were comparable across all datasets (Hallikar: ~18.5–21.9× coverage; mapping rate ~99.5–99.9%), indicating that differences in CNVR counts are not explained by sequencing depth or read quality.

• Second, CNVnator-derived raw CNV calls were compared across breeds. While variability in CNV counts was observed across all individuals—as expected for population-level CNV analysis - Hallikar samples did not show a consistent or systematic inflation relative to other breeds, and similar ranges of CNV counts were observed in other groups as well (please see the table below).

• Third, we examined CNVcaller intermediate outputs prior to merging. Hallikar also had similar number of initial window segments ~22k.

All samples were processed using identical parameters, normalization procedures, and filtering thresholds; therefore, downstream CNVR construction was performed uniformly across all breeds. Taken together, the consistency in sequencing metrics, the absence of systematic inflation in raw CNV counts across independent callers, and the uniform application of CNV filtering and merging criteria indicate that the higher number of Hallikar-specific CNVRs is not attributable to sequencing quality or methodological inconsistency but reflects variations in CNVs at individual animal level. For example, Table 3 shows that Hallikar had higher total and median CNVR lengths than the other breeds examined. This difference was primarily due to a greater number of duplication CNVRs identified in Hallikar. In contrast, the numbers of deletion and mixed CNVRs were comparable to those observed in the other breeds. These observations suggest that the higher number of Hallikar-specific CNVRs is unlikely to be an artefact of differences in CNV detection or filtering procedures.

CNVnator output for Hallikar against other breeds:

Sample Hallikar Kangayam Kdarm Red White Cattle

1 4,892 4,365 4,127 5,223

2 6,141 4,242 4,241 5,285

3 4,631 4,168 5,626 4,127

4 9,253 4,247 4,179 4,594

5 5,819 4,199 3,967 4,119

4. Please provide complete details of the enrichment analysis, including the background gene set, annotation source/version, and significance thresholds used.

Authors’ Response: We thank the Editor for requesting clarification regarding the enrichment analysis background and annotation details.Functional enrichment analysis was performed using ShinyGO. Since ShinyGO does not currently provide a dedicated annotation database for Bos indicus, we selected the closest available and well-annotated reference, Bos taurus (ARS-UCD1.2; Ensembl/STRING-db ID: btaurus_gene_ensembl; Taxonomy ID: 9913), for pathway and Gene Ontology term mapping. However, to ensure species-appropriate statistical inference, we defined the background gene set using all genes annotated in the Bos indicus NIAB GTF file derived from the reference genome (GCF_029378745.1).

https://ftp.ncbi.nlm.nih.gov/genomes/all/GCF/029/378/745/GCF_029378745.1_NIAB-ARS_B.indTharparkar_mat_pri_1.0/GCF_029378745.1_NIAB-ARS_B.indTharparkar_mat_pri_1.0_genomic.gtf.gz

This ensured that enrichment analyses were performed relative to the complete gene universe of the indicine genome used in this study, rather than relying on the default Bos taurus background. The list of background gene set used to perform enrichment analysis is now provided as Supporting Information File S3 in the revised manuscript. For significance thresholds, we applied the following criteria and is already included in the Methodology Section of the manuscript:

ALL_CNVRS_GOBP: FDR < 0.01 and fold enrichment (FE) > 2.5

Core_CNVRS_GOBP: FDR < 0.01 and FE > 2.5

HAL_CNVRS_GOBP: FDR < 0.01 and FE > 1.5

Journal Requirements:

If the reviewer comments include a recommendation to cite specific previously published works, please review and evaluate these publications to determine whether they are relevant and should be cited. There is no requirement to cite these works unless the editor has indicated otherwise.

Authors Response: Not Applicable

Please review your reference list to ensure that it is complete and correct. If you have cited papers that have been retracted, please include the rationale for doing so in the manuscript text, or remove these references and replace them with relevant current references. Any changes to the reference list should be mentioned in the rebuttal letter that accompanies your revised manuscript. If you need to cite a retracted article, indicate the article’s retracted status in the References list and also include a citation and full reference for the retraction notice.

Authors Response: The reference list was checked and found correct.

Attachment Submitted filename: 1.Mavunga et al_Authors_Response_Reviewers.docx

# Editor report: Decision Letter 2

**DOI:** 10.1371/journal.pone.0353468.r005

**Authors:** Tofazzal Md Rakib, Tofazzal Md Rakib, Tofazzal Md Rakib

24 Jun 2026

Genome-wide characterization of copy number variants and their functional relevance in indigenous draught cattle of South Asia

PONE-D-26-03707R2

Dear Dr. Periasamy,

We’re pleased to inform you that your manuscript has been judged scientifically suitable for publication and will be formally accepted for publication once it meets all outstanding technical requirements.

Within one week, you’ll receive an e-mail detailing the required amendments. When these have been addressed, you’ll receive a formal acceptance letter and your manuscript will be scheduled for publication.

An invoice will be generated when your article is formally accepted. Please note, if your institution has a publishing partnership with PLOS and your article meets the relevant criteria, all or part of your publication costs will be covered. Please make sure your user information is up-to-date by logging into Editorial Manager at Editorial Manager® and clicking the ‘Update My Information' link at the top of the page. For questions related to billing, please contact billing support.

If your institution or institutions have a press office, please notify them about your upcoming paper to help maximize its impact. If they’ll be preparing press materials, please inform our press team as soon as possible -- no later than 48 hours after receiving the formal acceptance. Your manuscript will remain under strict press embargo until 2 pm Eastern Time on the date of publication. For more information, please contact onepress@plos.org.

Kind regards,

Tofazzal Md Rakib, PhD

Academic Editor

PLOS One

Additional Editor Comments (optional):

Reviewers' comments:

# Editor report: Acceptance letter

**DOI:** 10.1371/journal.pone.0353468.r006

**Authors:** Tofazzal Md Rakib, Tofazzal Md Rakib, Tofazzal Md Rakib

PONE-D-26-03707R2

PLOS One

Dear Dr. Periasamy,

I'm pleased to inform you that your manuscript has been deemed suitable for publication in PLOS One. Congratulations! Your manuscript is now being handed over to our production team.

At this stage, our production department will prepare your paper for publication. This includes ensuring the following:

* All references, tables, and figures are properly cited

* All relevant supporting information is included in the manuscript submission,

* There are no issues that prevent the paper from being properly typeset

You will receive further instructions from the production team, including instructions on how to review your proof when it is ready. Please keep in mind that we are working through a large volume of accepted articles, so please give us a few days to review your paper and let you know the next and final steps.

Lastly, if your institution or institutions have a press office, please let them know about your upcoming paper now to help maximize its impact. If they'll be preparing press materials, please inform our press team within the next 48 hours. Your manuscript will remain under strict press embargo until 2 pm Eastern Time on the date of publication. For more information, please contact onepress@plos.org.

You will receive an invoice from PLOS for your publication fee after your manuscript has reached the completed accept phase. If you receive an email requesting payment before acceptance or for any other service, this may be a phishing scheme. Learn how to identify phishing emails and protect your accounts at https://explore.plos.org/phishing.

If we can help with anything else, please email us at customercare@plos.org.

Thank you for submitting your work to PLOS ONE and supporting open access.

Kind regards,

PLOS ONE Editorial Office Staff

on behalf of

Dr. Tofazzal Md Rakib

Academic Editor

PLOS One
